# Auriculocondylar syndrome 2 results from the dominant-negative action of *PLCB4* variants

**DOI:** 10.1242/dmm.049320

**Published:** 2022-04-29

**Authors:** Stanley M. Kanai, Caleb Heffner, Timothy C. Cox, Michael L. Cunningham, Francisco A. Perez, Aaron M. Bauer, Philip Reigan, Cristan Carter, Stephen A. Murray, David E. Clouthier

**Affiliations:** 1 Department of Craniofacial Biology, University of Colorado Anschutz Medical Campus, Aurora, CO 80045, USA; 2 The Jackson Laboratory, Bar Harbor, ME 04609, USA; 3 Departments of Oral and Craniofacial Sciences and Pediatrics, University of Missouri-Kansas City, Kansas City, MO 64108, USA; 4 University of Washington, Department of Pediatrics, Division of Craniofacial Medicine and Seattle Children's Craniofacial Center, Seattle, WA 98105, USA; 5 University of Washington, Department of Radiology and Seattle Children's Hospital, Seattle, WA 98105, USA; 6 Department of Biology, Villanova University, Villanova, PA 19085, USA; 7 Department of Pharmaceutical Sciences, University of Colorado Anschutz Medical Campus, Aurora, CO 80045, USA

**Keywords:** Craniofacial, G protein, Neural crest cell, CRISPR, Evolution, Mice

## Abstract

Auriculocondylar syndrome 2 (ARCND2) is a rare autosomal dominant craniofacial malformation syndrome linked to multiple genetic variants in the coding sequence of phospholipase C β4 (*PLCB4*). PLCB4 is a direct signaling effector of the endothelin receptor type A (EDNRA)-Gq/11 pathway, which establishes the identity of neural crest cells (NCCs) that form lower jaw and middle ear structures. However, the functional consequences of *PLCB4* variants on EDNRA signaling is not known. Here, we show, using multiple signaling reporter assays, that known *PLCB4* variants resulting from missense mutations exert a dominant-negative interference over EDNRA signaling. In addition, using CRISPR/Cas9, we find that F_0_ mouse embryos modeling one *PLCB4* variant have facial defects recapitulating those observed in hypomorphic *Ednra* mouse models, including a bone that we identify as an atavistic change in the posterior palate/oral cavity. Remarkably, we have identified a similar osseous phenotype in a child with ARCND2. Our results identify the disease mechanism of ARCND2, demonstrate that the *PLCB4* variants cause craniofacial differences and illustrate how minor changes in signaling within NCCs may have driven evolutionary changes in jaw structure and function.

This article has an associated First Person interview with the first author of the paper.

## Abstract (precis)

**Summary:** PLCB4 variants associated with auriculocondylar syndrome act in a dominant-negative manner to cause craniofacial defects that include both homeotic and atavistic changes in mice and humans.

## INTRODUCTION

The maxilla, mandible and intervening bone (jugal/zygoma and portions of the squamosal bone) are derived from neural crest cells (NCCs) that reside in the first pharyngeal arch (Abe et al., 2007; Chai et al., 2000). First arch NCCs initially possess developmental plasticity to form either elements of the upper or lower jaw (Barron et al., 2011; Minoux et al., 2017; Sato et al., 2008; Tavares and Clouthier, 2015), with those in the mandibular region of first arch adopting a lower jaw identity through cell-autonomous endothelin receptor type A (EDNRA) signaling (Clouthier et al., 2003; Ferguson et al., 2000; Ozeki et al., 2004; Ruest et al., 2004). Upon binding of its ligand, endothelin 1 (EDN1) (Arai et al., 1990; Davenport et al., 2016), EDNRA activates the Gq/11 class of heterotrimeric G proteins in NCCs (Dettlaff-Swiercz et al., 2005; Ivey et al., 2003; Offermanns et al., 1998). Gq/11 subsequently activates phospholipase C β (PLCB) isoforms, which, in turn, convert phosphatidylinositol 4,5-bisphosphate (PIP2) into inositol 1,4,5-triphosphate (IP3) and diacylglycerol (DAG) (Kadamur and Ross, 2013), resulting in changes in gene expression that drive NCC patterning. The requirement of EDNRA signaling in lower jaw development appears conserved in all jawed vertebrates (Clouthier et al., 2013), including humans (Pritchard et al., 2020), illustrating that it is a fundamental mechanism that drives facial development.

There is increasing evidence that a number of gene variants can disrupt the EDNRA-Gq/11-PLCB4 signaling axis and give rise to phenotypically distinct human craniofacial disorders. Oro-oto-cardiac syndrome (OOCS) and mandibulofacial dysostosis with alopecia (MDFA; MIM 616367) are caused by variants in *EDNRA* (MIM 131243), although the variants affect EDNRA function differently. The OOCS-associated disease variant EDNRA p.Gln381Pro disrupts receptor-Gq/11 association and abrogates signaling, leading to cardiac and craniofacial defects resembling the mouse *Ednra* knockout phenotype, including neonatal lethality (Pritchard et al., 2020). The MFDA-associated disease variants EDNRA p.Tyr129Phe (c.386A>T) and EDNRA p.Glu303Lys (c.907G>A) likely reduce receptor-EDN1 binding affinity and increase receptor-EDN3 binding affinity, leading to ectopic EDNRA signaling in the maxillary prominence (Gordon et al., 2015). These changes result in defects in the mandible and maxilla, although cardiovascular development is unaffected.

Distinct from OOCS and MFDA, auriculocondylar syndrome (ARCND) is a genetically heterogenous disorder primarily caused by variants in three different signaling proteins that function within the EDNRA-Gq/11-PLCB signaling pathway. ARCND-associated variants in *GNAI3* (MIM 139370), *PLCB4* (MIM 600810) and *EDN1* (MIM 131240) are classified as ARCND1 (MIM 602483), ARCND2 (MIM 614669) and ARCND3 (MIM 615706), respectively, with all three types characterized by micrognathia, temporomandibular joint ankylosis and a stereotypical outer ear deformity called question mark ears (QME) (Gordon et al., 2013a,b; Nabil et al., 2020; Rieder et al., 2012; Romanelli Tavares et al., 2015). Although the ARCND phenotype resembles OOCS and the *Ednra* mutant mouse phenotype, cardiovascular defects and neonatal lethality are not observed. The reason for this difference is still unknown.

The autosomal dominant inheritance and nature of the protein-coding mutations of ARCND1 (*GNAI3*) and ARCND2 (*PLCB4*) have led researchers to hypothesize that dominant-negative interference of the EDNRA signaling pathway is the underlying disease mechanism (Clouthier et al., 2013; Gordon et al., 2013b; Rieder et al., 2012). Indeed, it was demonstrated that *GNAI3* variants encode dominant-negative mutants of GNAI3 that form a nonproductive complex with EDNRA, abrogating EDNRA-mediated Gq/11 activation (Marivin et al., 2016). The disease mechanism for ARCND2 remains to be elucidated.

In this study, we examined the disease mechanism of ARCND2-associated PLCB4 variants using *in vitro* and *in vivo* approaches. Using a variety of signaling reporter assays in cell culture, we show that PLCB4 variants interfere with EDNRA-Gq/11-mediated activity of wild-type PLCB in a partially dominant-negative manner, with this interference blocked by disrupting the Gq/11-PLCB4 binding interface. Furthermore, using CRISPR/Cas9 gene editing to model ARCND2 in F_0_ mice, we find that insertion of a human *PLCB4* variant produces defects resembling those in mouse models in which EDNRA signaling is reduced, but not lost, suggesting that ARCND is caused by a partial reduction in EDNRA signaling. These F_0_ CRISPR embryos have also allowed us to re-interpret the novel craniofacial differences observed in individuals with ARCND2. Importantly, one of the observed changes, the formation of an osseous strut on the lateral pterygoids, resembles the posterior projection of the pterygoid observed in some non-mammalian amniotes. This likely atavistic change resulting from low-level EDNRA signaling underscores the flexibility and sensitivity of NCCs to undergo significant evolutionary change in response to minor changes in cell signaling pathways.

## RESULTS

**Fig. 1.**
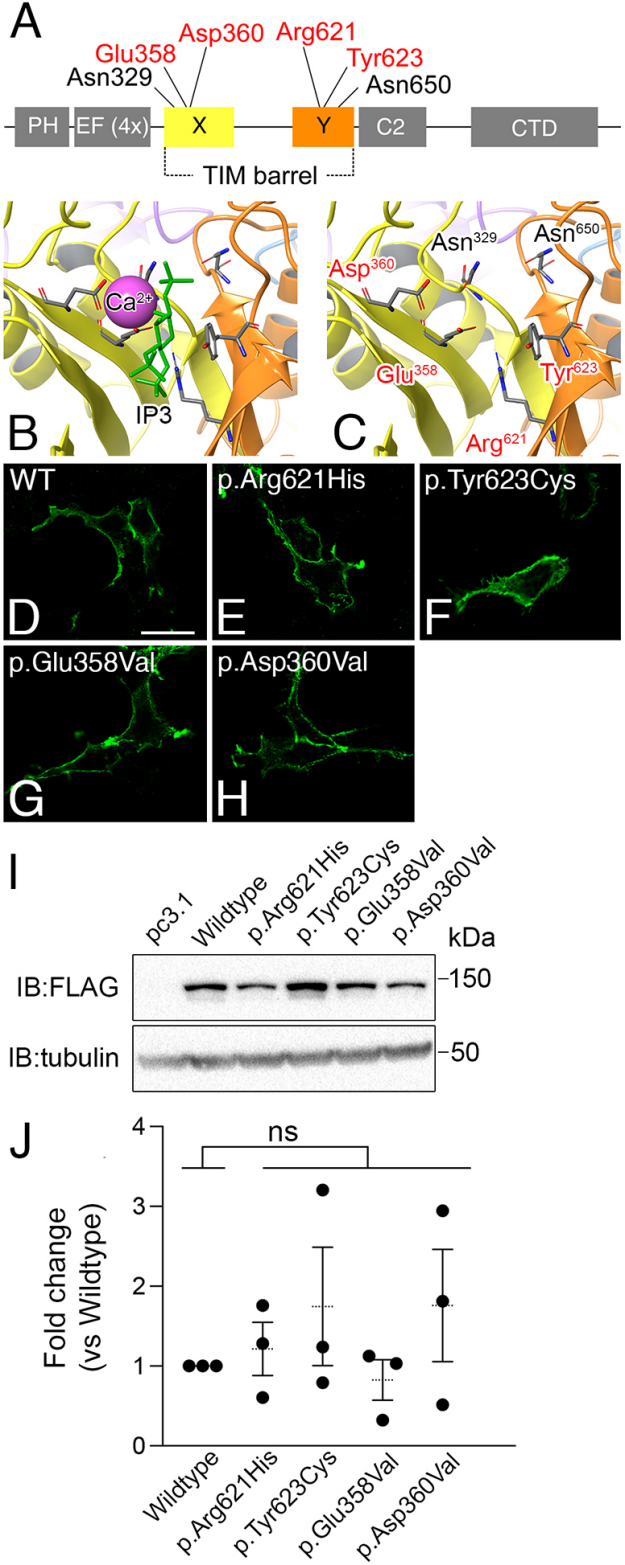
**ARCND2-associated variants cause missense mutations in PLCB4 active site residues.** (A) Protein domains of PLCB4. ARCND2 variants affect residues in the X and Y domains that form the catalytic pocket. Residues examined in this study are highlighted in red in A and C. (B,C) The active site in the PLCB4 homology model, with yellow and orange structures representing the X and Y domains, respectively. The calcium ion (Ca^2+^, purple) and IP3 molecule (green) are superimposed on the active site (B), and are surrounded by residues affected by ARCND2 variants (in red in C). (D-H) Representative immunofluorescence confocal microscopy images of HEK293T cells transfected with Myc-FLAG-tagged wild-type PLCB4 or PLCB4 mutants and visualized with an Alexa Fluor 488-labeled anti-Myc antibody. Scale bar: 20 µm. (I) Representative western blot showing relative expression levels of Myc-FLAG-tagged wild-type PLCB4 or PLCB4 mutants transfected into HEK293T cells. (J) Quantification of PLCB4 mutant expression in western blot analysis, shown as fold difference relative to wild-type expression. Each data point represents a biological replicate (*n*=3). Statistical significance versus wild type was calculated using Prism and an unpaired two-tailed *t*-test (p.Arg621His, *P*=0.56; p.Tyr623Cys, *P*=0.37; p.Glu358Val, *P*=0.53; p.Asp360Val, *P*=0.34). CTD, C-terminal coiled-coil domain; EF (4X), four tandem copies of the EF-Hand motif; IB, immunoblot; kDa, kilo Dalton; ns, not significant. PH, pleckstrin homology domain.

The majority of *PLCB4* variants identified to date in ARCND2 cause missense mutations in the conserved catalytic pocket of PLCB4 (Gordon et al., 2013b; Nabil et al., 2020; Rieder et al., 2012), where PIP2 is hydrolyzed into DAG and IP3 (Fig. 1A-C) (Kadamur and Ross, 2013). Given the functional importance of the affected residues and the autosomal dominant inheritance of ARCND2, we and others have hypothesized that these PLCB4 mutants exert a dominant-negative effect on wild-type PLCB isoforms likely by forming a nonproductive enzyme-substrate complex with PIP2, leading to insufficient activation of the EDNRA-Gq/11-PLCB signaling pathway during craniofacial patterning (Clouthier et al., 2013; Gordon et al., 2013b; Rieder et al., 2012). To test this, we first created expression plasmids for ARCND2 variants of PLCB4: p.Arg621His (c.1862G>A; Gordon et al., 2013b; Rieder et al., 2012), p.Tyr623Cys (c.1868A>G; Rieder et al., 2012), pGlu358Val (c.1073A>T; Gordon et al., 2013b; Shkalim et al., 2008) or p.Asp360Val (c.1079A>T; Gordon et al., 2013b; Greig et al., 2012). When transfected into HEK293T cells, confocal microscopy showed that the extent of membrane fluorescence of the variants was similar to that of wild-type PLCB4 (Fig. 1D-H). Furthermore, quantitative analysis of western blots from three different transfection experiments found no statistical difference in the expression level of the constructs (Fig. 1I,J).

**Fig. 2.**
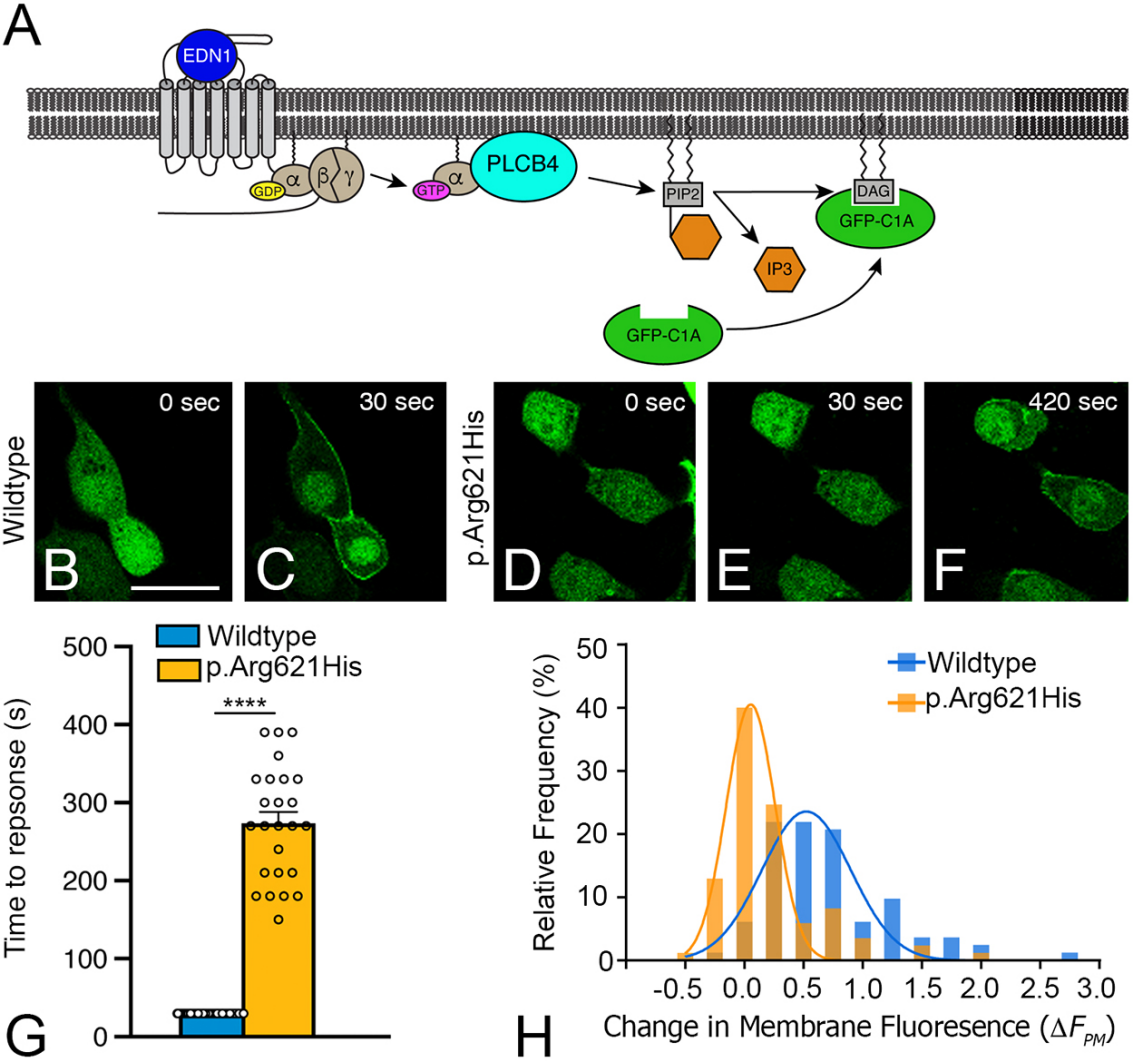
**PLCB4 p.Arg621His attenuates EDNRA signaling.** (A) Schematic showing endothelin 1 (EDN1)/endothelin A receptor (EDNRA) signaling triggering PLCB4-mediated PIP2 hydrolysis. This results in cytoplasm-to-plasma membrane translocation of the DAG sensor GFP-C1A. (B-F) Representative time-lapse images of the GFP translocation assay in HEK293T cells transfected with GFP-C1A, EDNRA and wild-type PLCB4 or PLCB4 p.Arg621His. Time relative to EDN1 addition is noted. Scale bar: 20 µm. (G) Quantification of the translocation response time observed in individual cells (open circles), with only responding cells used for data analysis (*n*=90 for wild-type PLCB4-tranfected cells and *n*=25 for PLCB4 p.Arg621His-transfected cells). Error bars represent s.e.m. Statistical significance was calculated using Prism and an unpaired two-tailed *t*-test (*P*<0001). ****P<0.0001. (H) Quantification of the translocation response profiles for cell populations expressing wild-type PLCB4 or PLCB4 p.Arg621His. The translocation response was quantified for individual cells by measuring the relative increase in membrane fluorescence, expressed as Δ*F*_pm_ on the *x*-axis. The *y*-axis represents the percentage of total cells that elicited the indicated Δ*F*_pm_ value. α, G protein α subunit q (Gαq); β, G protein β subunit; DAG, diacylglycerol; γ, G protein γ subunit; IP3, inositol triphosphate.

We next tested whether overexpression of a representative PLCB4 variant, p.Arg621His, would interfere with endogenous PLCB isoforms in HEK293T cells. PLCB activity was assessed using time-lapse imaging and the fluorescent reporter GFP-C1A that translocates from the cytoplasm to plasma membrane upon DAG production (Oancea et al., 1998) (Fig. 2A). Of the cells transfected with wild-type PLCB4, in which EDN1 addition elicited a translocation response, membrane fluorescence was observed within the first imaging frame after EDN1 addition [30±0 s (s.e.m.)] (Fig. 2B,C,G). Cells transfected with empty vector responded to EDN1 similarly, likely due to endogenous PLCB (Fig. S1). In contrast, in cells transfected with PLCB4 p.Arg621His, EDN1 addition elicited a significantly delayed translocation response, with an average response time of 270±70 s (s.e.m.) (*P*<0.0001) (Fig. 2D-G). We then quantified the difference in the translocation response by calculating the relative increase in membrane fluorescence (Δ*F*_pm_; see Materials and Methods) for individual cells in each population, plotting the Δ*F*_pm_ values in a histogram, and fitting the data to a Gaussian curve. While the mean Δ*F*_pm_ value for wild-type PLCB4-expressing cells was 0.52±0.038 (s.e.m.) (Fig. 2H, blue bars), the mean Δ*F*_pm_ value for PLCB4 p.Arg621His-expressing cells was 0.053±0.015 (s.e.m.) (Fig. 2H, yellow bars), with lower Δ*F*_pm_ values indicating a weaker translocation response. The difference in the distribution curve was statistically significant (*P*<0.0001). Together, these results indicate that PLCB4 p.Arg621His interferes with endogenous PLCB activity in a dominant-negative manner, attenuating both the extent and latency of activation.

### BRET analysis of PLCB4 variants supports a dominant-negative mechanism

**Fig. 3.**
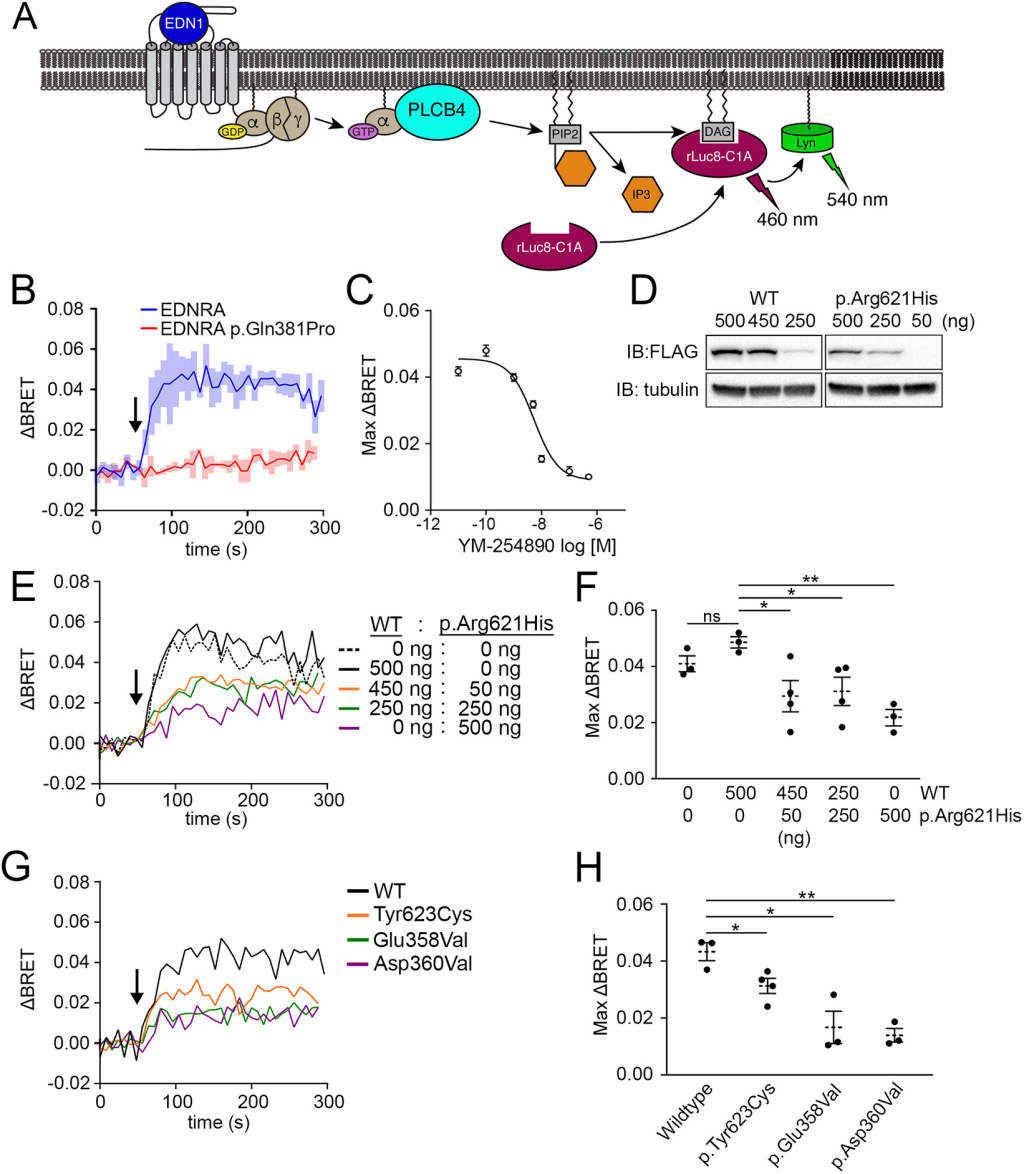
**ARCND2-associated PLCB4 mutants exert dominant-negative interference of the EDNRA signaling pathway.** (A) Schematic showing how BRET is achieved in response to EDN1. Upon translocation-induced proximity, luminescence (460 nm) from the rLuc8-C1A BRET donor excites the Lyn-Venus BRET acceptor, resulting in fluorescence emission (540 nm) (ΔBRET; see Materials and Methods). (B) ΔBRET response after EDN1 stimulation (arrow) in HEK293T cells co-transfected with rLuc8-C1A, Lyn-Venus and wild-type EDNRA or a loss-of-function EDNRA mutant (p.Gln381Pro). ΔBRET traces are an average of three experiments. Shaded boxes represent s.e.m. (C) The Gq/11-specific inhibitor YM-254890 attenuates the EDN1-stimulated maximum ΔBRET response in cells transfected with BRET sensor components and EDNRA. Each point represents an average of at least three experiments. (D) Representative western blots of cells transfected with the indicated amount (nanograms; ng) of plasmid. (E) ΔBRET response after EDN1 stimulation (arrow) in cells co-transfected with BRET sensor components, EDNRA and differing amounts of wild-type PLCB4 and PLCB4 p.Arg621His. ΔBRET traces are an average of three (0:0, 500:0 and 0:500), four (450:50) or five (250:250) individual experiments. (F) Quantification of the maximum ΔBRET response in E. Data points are individual experiments with *n* values listed in E. Error bars represent s.e.m. Statistical significance was calculated using Prism and an unpaired two-tailed *t*-test (mock transfection versus 500:0, *P*=0.093; 500:0 versus 450:50, *P*=0.0372; 500:0 versus 250:250, *P*=0.037; 500:0 versus 0:500, *P*=0.0017). (G) ΔBRET response after EDN1 stimulation (arrow) in cells co-transfected with BRET sensor components, EDNRA and wild-type PLCB4 or the indicated PLCB4 mutant. Traces are an average of three experiments, except for p.Glu358Val (*n*=4). (H) Quantification of the maximum ΔBRET response in G. Data points are individual experiments with *n* values listed in G. Error bars represent s.e.m. Statistical significance versus wild type was calculated using Prism and an unpaired two-tailed *t*-test (p.Tyr623Cys, *P*=0.032; p.Glu358Val, *P*=0.015; p.Asp360Val, *P*=0.0018). *P<0.05; **P<0.01. IB, immunoblot antibody; ns, not significant.

To investigate the dominant-negative effect of ARCND2 variants using a more quantitative approach, we designed a bystander Bioluminescence Resonance Energy Transfer (BRET) assay (Namkung et al., 2016) using the principle of the GFP-C1A translocation assay. The PKCγ C1A domain (C1A) was tagged with Renilla Luciferase 8 (rLuc8) to create a rLuc8-C1A BRET donor. When used together with a plasma membrane-localized acceptor protein, Lyn-Venus (Gulyás et al., 2017), the presence of DAG elicits a BRET signal by translocation-induced proximity of rLuc8-C1A and Lyn-Venus (Fig. 3A). A previous study demonstrated that a Fluorescence Resonance Energy Transfer-based version of this assay accurately reports DAG production mediated by Gq-coupled receptors (Falkenburger et al., 2013).

To verify the specificity of the BRET signal using this approach, cells were co-transfected with rLuc8-C1A and Lyn-Venus along with EDNRA or a loss-of-function *EDNRA* variant [EDNRA p.Gln381Pro (c.1142A>C; Pritchard et al., 2020)]. Subsequent EDN1 treatment produced a ΔBRET response from cells expressing EDNRA but not from cells expressing EDNRA p.Gln381Pro (Fig. 3B). Furthermore, pretreating cells with a Gq/11-specific small molecule inhibitor YM-254890 (Nishimura et al., 2010; Taniguchi et al., 2003) attenuated the ΔBRET response in a concentration-dependent manner (Fig. 3C). These data demonstrate that the EDN1-stimulated ΔBRET response is mediated by the EDNRA-Gq/11 signaling pathway.

Using this assay, we examined the functional impact of PLCB4 p.Arg621His on EDN1/EDNRA/Gq-mediated PLCB activity. While EDN1 treatment of cells transfected with 500 ng of empty vector or wild-type PLCB4 elicited similar maximum ΔBRET responses of 0.041±0.0023 (s.e.m.) and 0.049±0.0014 (s.e.m.), respectively (Fig. 3D-F), cells transfected with 500 ng of PLCB4 p.Arg621His elicited a relatively lower maximum ΔBRET response of 0.022±0.0019 (s.e.m.) (Fig. 3D-F), indicating that PLCB4 p.Arg621His interferes with the activity of endogenous PLCB isoforms. The maximum ΔBRET response was also attenuated in cells co-transfected with a 1:1 [250 ng:250 ng, 0.031±0.0044 (s.e.m.)] or 9:1 [450 ng:50 ng, 0.029±0.0056 (s.e.m.)] ratio of plasmid for wild-type PLCB4 and PLCB4 p.Arg621His, respectively (Fig. 3D-F). These results illustrate that PLCB4 p.Arg621His interferes with PLCB activity even when it is expressed at a sub-stoichiometric level relative to wild-type PLCB4, exemplifying a characteristic of a dominant-negative protein. We confirmed that other ARCND2 variants, PLCB4 p.Tyr623Cys [0.031±0.0023 (s.e.m.)], PLCB4 p.Glu358Val [0.017±0.0047 (s.e.m.)] and PLCB4 p.Asp360Val [0.00.014±0.0019 (s.e.m.)], similarly behaved like dominant-negative proteins. Cells transfected with plasmid (500 ng) for any one of these variants resulted in an attenuated ΔBRET response (Fig. 3G,H).

### The dominant-negative effect of PLCB4 p.Arg621His is abolished by blocking Gq/11-mediated activation

**Fig. 4.**
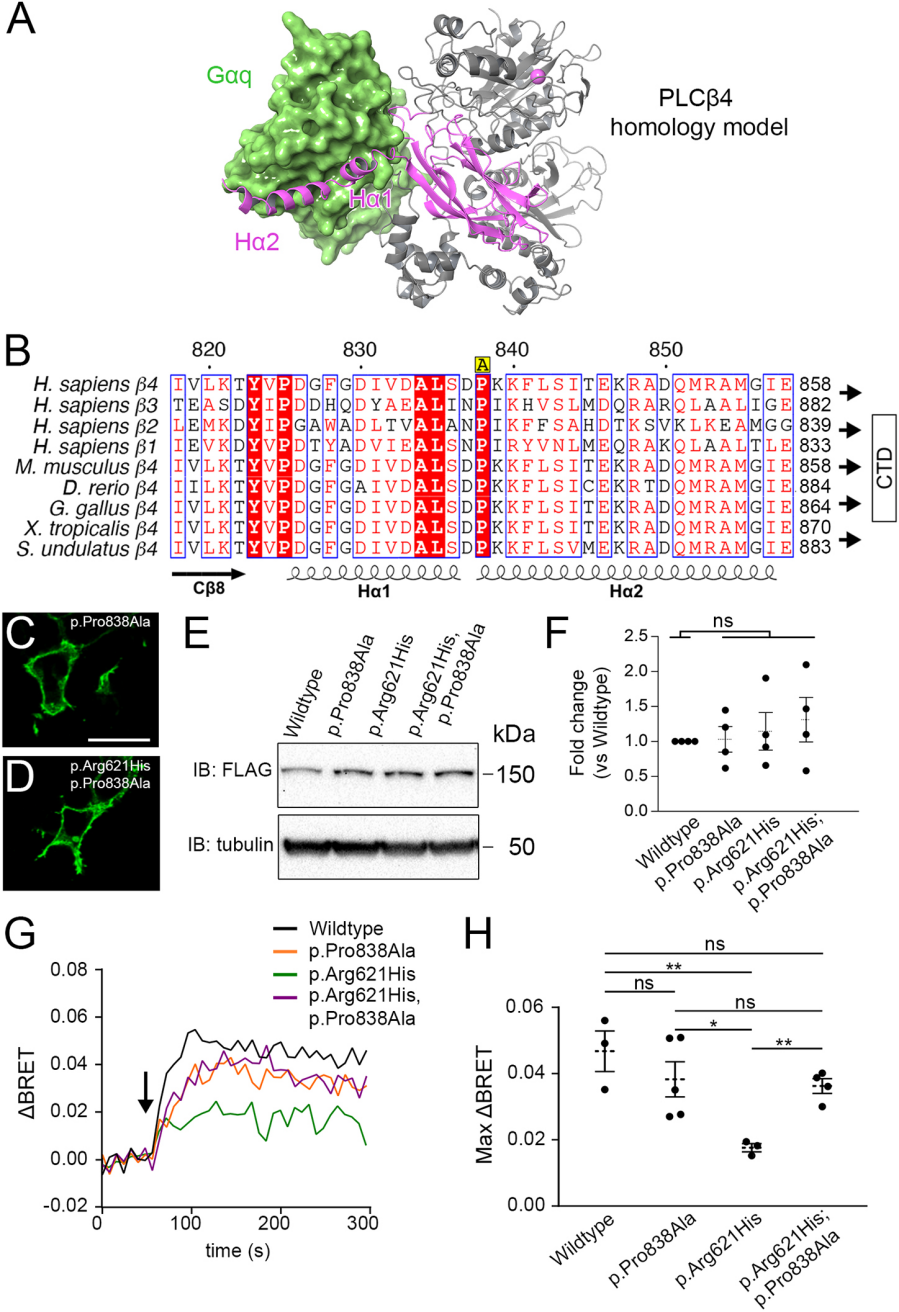
**The dominant-negative action of PLCB4 p.Arg621His is alleviated by disrupting the Gq-PLCB4 interaction interface.** (A) Homology model of PLCB4 (gray and purple ribbons) shown interacting with a GTP-bound Gαq crystal structure (green) and containing a calcium ion (Ca^2+^, purple). The Hα1/Hα2 helix-turn-helix motif on the C2 domain C terminus (purple) of PLCB4 binds to a shallow cavity in Gαq. (B) Sequence alignment of the Hα1 and Hα2 helix-turn-helix motif for human PLCB isoforms and PLCB4 from different species isoforms that are required for Gq-mediated activation (Waldo et al., 2010). Red boxes with white characters represent highly conserved residues; red characters represent residues with equivalent physicochemical properties; blue frames highlight highly conserved regions. The conserved proline at position 838 was mutagenized in this study to an alanine (p.Pro838Ala in PLCB4) and is denoted in yellow above the sequence by an ‘A’. (C,D) Representative immunofluorescence confocal microscopy images of cells transfected with indicated PLCB4 mutants and visualized with an Alexa Fluor 488-labeled anti-Myc antibody. Scale bar: 20 µm. (E) Representative western blot showing expression levels of wild-type PLCB4 and PLCB4 mutants transfected in HEK293T cells. (F) Quantification of PLCB4 variant expression in western blot analysis, expressed as fold difference relative to wild-type expression (*n*=4). Each data point represents a biological replicate. Statistical significance versus wild type was calculated using Prism and an unpaired two-tailed *t*-test (p.Pro838Ala, *P*=0.88; p.Arg621His, *P*=0.61; p.Arg621His;p.Pro838Ala, *P*=0.37). (G) EDN1-stimulated (arrow) ΔBRET response in cells co-transfected with BRET sensor components, EDNRA and wild-type PLCB4 or indicated PLCB4 mutants. Traces are an average of at least three experiments (*n*=3 for wild type and p.Arg621His, *n*=4 for p.Arg621His;p.Pro838Ala and *n*=5 for p.Pro838Ala). (H) Quantification of maximum ΔBRET response in G. Each data point represents a biological replicate with *n* values given in G. Error bars represent s.e.m. Statistical significance was calculated using Prism and an unpaired two-tailed *t*-test for the following comparisons. Wild type versus: p.Pro838Ala, *P*=0.35; p.Arg621His, *P*=0.0096; p.Arg621His;p.Pro838Ala, *P*=0.13. p.Pro838Ala versus: p.Arg621His, *P*=0**.**027; p.Arg621His;p.Pro838Ala, *P*=0.76. p.Arg621His versus p.Arg621His;p.Pro838Ala, *P*=0.0012. *P<0.05, **P<0.01; ns, not significant.

PLCB isoforms are activated by Gq/11 through a critical Gq/11-PLCB binding interface that is facilitated by a conserved Hα1/Hα2 helix-turn-helix motif on the C2 domain C terminus in PLCB isoforms (Waldo et al., 2010) (Fig. 4A). This region of PLCB isoforms is highly conserved among a wide variety of vertebrates, including *Sceloporus undulatus* (the Eastern fence lizard) (Fig. 4B). In principle, blocking the activation mechanism of a PLCB4 mutant would diminish its dominant-negative effect. We tested this by introducing a point mutation, p.Pro838Ala (c.2512C>G), which should disrupt a conserved PLCB-Gq/11-binding interface that is required for PIP2 hydrolysis (Waldo et al., 2010), to wild-type PLCB4 and *in cis* with PLCB4 p.Arg621His (Fig. 4B). After transfecting the two variants, confocal microscopy showed that the extent of membrane fluorescence of PLCB4 p.Pro838Ala (Fig. 4C) and PLCB4 p.Arg621His;p.Pro838Ala (Fig. 4D) was similar. Furthermore, quantitative analysis of western blots from four different transfection experiments found no statistical difference in the expression of the two constructs compared with wild-type PLCB4 and PLCB4 p.Arg621His (Fig. 4E,F). In our BRET assay, although the maximum ΔBRET response after EDN1 addition was slightly lower in cells expressing PLCB4 p.Pro838Ala [(0.038±0.0040 (s.e.m.)] compared with wild-type PLCB4 [(0.047±0.0043 (s.e.m.)], the difference was not statistically significant (Fig. 4G,H). In contrast, the maximum ΔBRET response was approximately twofold greater in cells expressing PLCB4 p.Arg621His;p.Pro838Ala [(0.036±0.0016 (s.e.m.)] compared with cells expressing PLCB4 p.Arg621His. [0.018±0.00089 (s.e.m.)] (Fig. 4G,H), indicating that the p.Pro838Ala mutation diminished the dominant-negative effect of PLCB4 p.Arg621His. The difference between wild-type PLCB4 and PLCB4 p.Arg621His;p.Pro838Ala was not statistically significant. These results illustrate that the dominant-negative effect of PLCB4 mutants is dependent on Gq/11-mediated activation.

### PLCB4 dominant-negative variants interfere with downstream signaling events

**Fig. 5.**
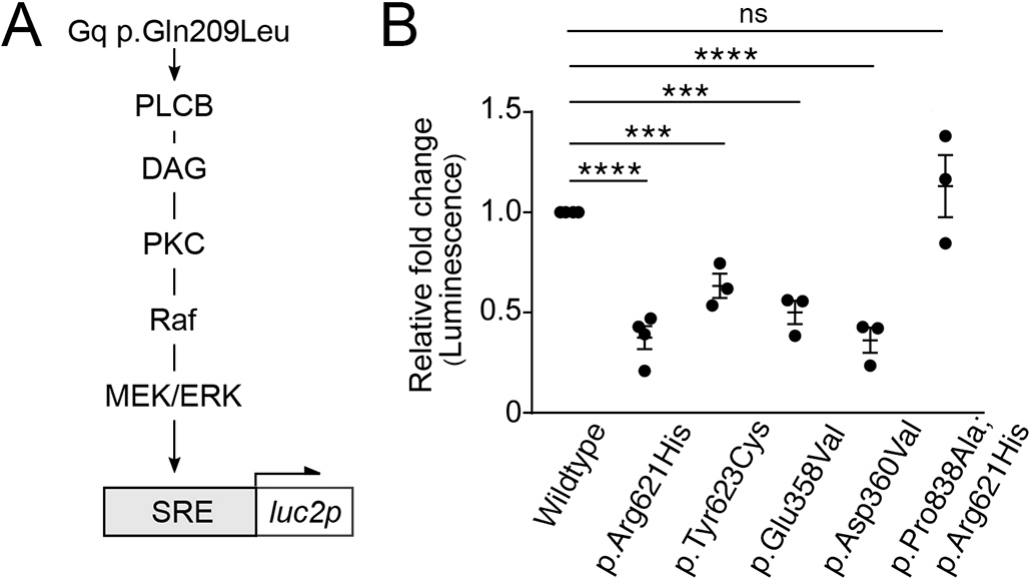
**ARCND2-associated PLCB4 mutants interfere with SRE activity in a dominant-negative manner.** (A) Schematic of the signaling pathway that stimulates SRE:*luc2p* transcription. Gq p.Gln209Leu activates the Raf-MEK/ERK pathway through PLCB, resulting in activation of the serum response element (SRE). (B) Relative luminescence from cells co-transfected with SRE:*luc2p*, Gq p.Gln209Leu and wild-type or mutant PLCB4. Luminescence is expressed as the fold change relative to cells expressing wild-type PLCB4. Data points are individual experiments [*n*=3 for all samples except wild type and p.Arg621His (*n*=4)]. Error bars represent s.e.m. Significance versus wild type was calculated using Prism and an unpaired two-tailed *t*-test (p.Arg621His, *P*<0.0001; p.Tyr623Cys, *P*=0.0008; p.Glu358Val, *P*=0.0002; p.Asp360Val, *P*<0.0001; p.Pro838Ala;p.Arg621His, *P*=0.36). ***P<0.001, ****P<0.0001; ns, not significant.

The production of DAG by the EDNRA-Gq/11-PLCB signaling pathway subsequently activates the MEK/ERK pathway (Cai et al., 1997; Janknecht et al., 1993) (Fig. 5A), which is involved in lower jaw development. E18.5 *Erk2^fl/fl^;Wnt1-Cre* embryos have significant lower jaw defects (Parada et al., 2015), further highlighting the significance of this pathway. To determine whether dominant-negative PLCB4 variants disrupt the MEK/ERK pathway, we used a serum response element (SRE)-controlled transcriptional reporter, SRE:*luc2p*, that expresses luciferase upon activation of the MEK/ERK pathway (Cheng et al., 2010) (Fig. 5A). HEK293 cells were co-transfected with a constitutively active Gq mutant Gq p.Gln209Leu (c.626A>T) (Kalinec et al., 1992), which stimulates SRE activity via the MEK/ERK pathway in a receptor-independent manner, and either wild-type PLCB4 or PLCB4 mutants. SRE activity was reduced in PLCB4 mutant-expressing cells compared with wild-type PLCB4-expressing cells, with a reduction in SRE activity of 37.6% (PLCB4 p.Arg621His), 63.4% (PLCB4 p.Tyr623Cys), 50.1% (PLCB4 p.Glu358Val) and 36.2% (PLCB4 p.Asp360Val) (Fig. 5B). The difference in SRE activity was not statistically significant between cells expressing wild-type PLCB4 and PLCB4 p.Arg621His;p.Pro838Ala compound mutant (Fig. 5B). These findings illustrate that dominant-negative interference of the initial secondary messenger response (IP3 and DAG production) by PLCB4 mutants leads to the abatement of downstream signaling and gene expression events.

### An ARCND2-associated *PLCB4* mutation recapitulates hypomorphic *Ednra* phenotypes in mice

We next examined whether an ARCND2-associated variant is sufficient to recapitulate the ARCND phenotype in mice. To do this, CRISPR/Cas9 and homology-directed repair (HDR) was used to generate knock-in ‘founder’ (F_0_) perinatal embryos harboring the orthologous variant (c.1862G>A; p.Arg621His) in *Plcb4*. Of the 69 embryos harvested at E18.5, we identified three embryos harboring at least one knock-in allele; one was homozygous for the knock-in allele (*Plcb4^KI/KI^*) and the other two were compound heterozygotes harboring the knock-in allele and an insertion/deletion (indel) allele generated by non-homologous end joining (NHEJ) (*Plcb4^Indel/KI^*). Five other embryos were heterozygous for an indel allele (*Plcb4^wt/Indel^*). Embryos harboring at least one knock-in allele (*n*=3) or only indel alleles (*n*=5) were scanned using micro-computed tomography (µCT), and embryos harboring a knock-in allele were subsequently processed for bone and cartilage staining with Alizarin Red and Alcian Blue.

**Fig. 6.**
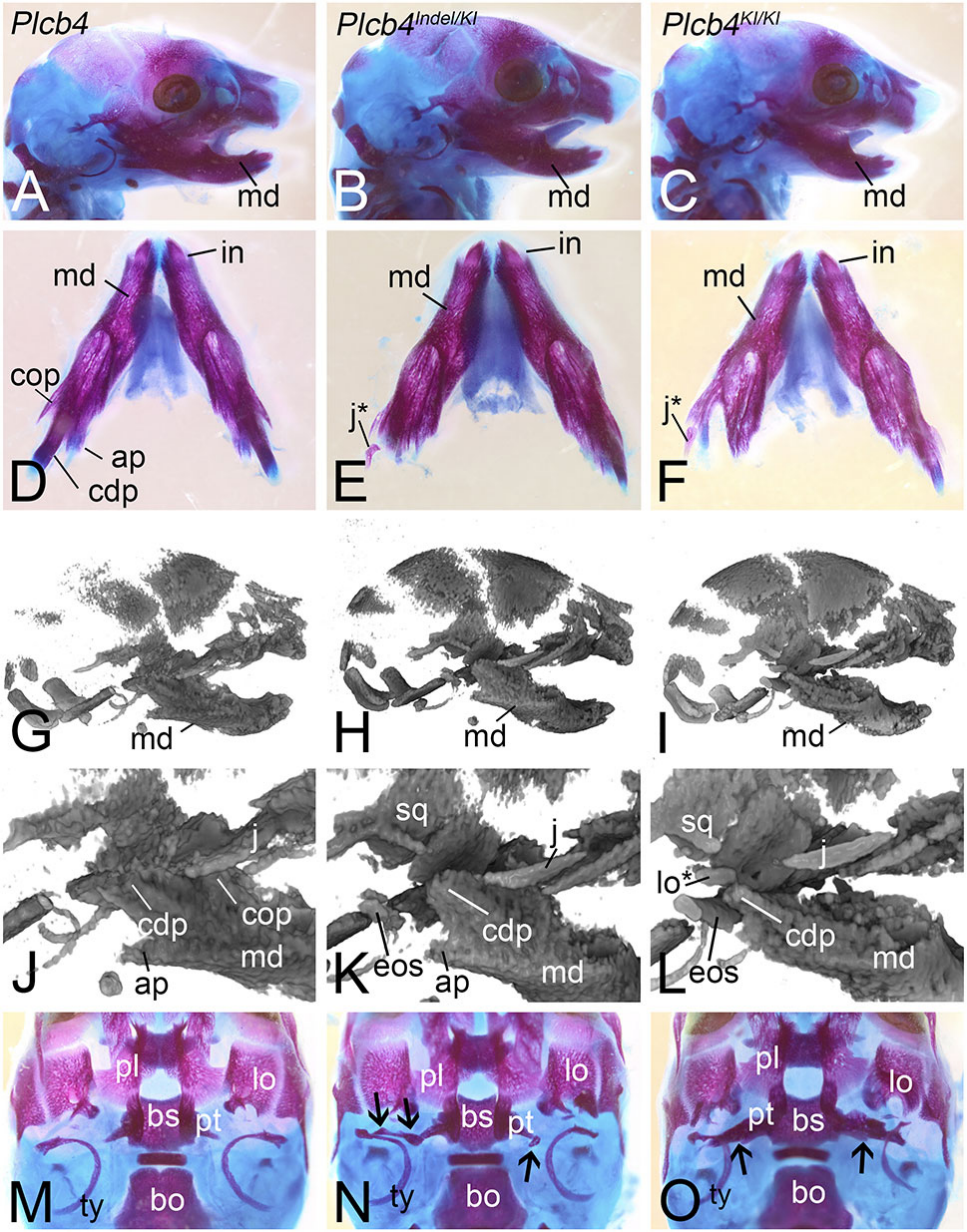
**F0 CRISPR mouse model of ARCND.** (A-O) Skulls from E18.5 embryos following Alizarin Red and Alcian Blue staining (A-F,M-O) or µCT scanning (G-L) of wild-type *Plcb4* (A,D,G,J,M), *Plcb4^Indel/KI^* (B,E,H,K,N) and *Plcb4^KI/KI^* (C,F,I,L,O) embryos. Black arrows in N and O indicate the presence of an ectopic osseous strut (eos). ap, angular process; bo, basioccipital bone; bs, basisphenoid bone; cdp, condylar process; cop, coronoid process; in, incisor; j, jugal; j*, duplicated jugal; lo, lamina obturans; lo*, duplicated lamina obturans; md, mandible; pl, palatine; pt, pterygoid; sq, squamosal; ty, tympanic ring bone.

All F_0_ embryos harboring at least one knock-in (KI) allele (*Plcb4^KI/KI^* and *Plcb4^Indel/KI^*) had several defects in head skeletal structures previously observed in hypomorphic *Ednra* mouse models (Clouthier et al., 2003; Ruest and Clouthier, 2009; Tavares et al., 2012). Compared with control (*Plcb4^wt/wt^*) embryos (Fig. 6A,D; *n*=3), the proximal mandible of *Plcb4^Indel/KI^* and *Plcb4^KI/KI^* embryos was disorganized, with absent or hypoplastic mandibular processes (Fig. 6B,C,E,F). This included reduction in the size of the condylar process and reduction or loss of the coronoid and angular processes (Fig. 6E,F). In addition, a small bone was attached to the proximal mandible by a fibrous suture that we have previously identified as a duplication of the jugal bone of the zygomatic arch that occurs in hypomorphic *Ednra* mouse models (Clouthier et al., 2003; Ruest and Clouthier, 2009; Tavares et al., 2012). µCT scans performed before bone and cartilage staining provided additional resolution of these changes, including dysplasia or absence of the coronoid and angular processes in *Plcb4^KI/KI^* embryos (Fig. 6H,I,K,L) compared with a control embryo (Fig. 6G,J). No overt changes in skull structure were observed by µCT in embryos harboring only indels (*Plcb4^wt/Indel^*, *n*=5) (data not shown) or in embryos with unmodified alleles (*n*=3) (data not shown). The normal craniofacial phenotype of *Plcb4^wt/Indel^* embryos is consistent with *Plcb4^+/−^* and *Plcb4^−/−^* mice, which have no reported changes in skull structures (Jiang et al., 1996). These results indicate that a single *Plcb4^KI^* allele is sufficient to cause craniofacial defects.

In addition to the duplicated jugal bone, we observed an additional defect in the posterior roof of the oral cavity is often associated with reduced (though not absent) EDNRA signaling (Clouthier et al., 2003; Ruest and Clouthier, 2009). Ectopic osseus struts of variable size and shape extended mediolaterally from the lateral pterygoid bones (Fig. 6K,L,N,O) to middle ear structures. The bone struts were larger in the *Plcb4^KI/KI^* embryo compared with the *Plcb4^Indel/KI^* embryo (Fig. 6N,O), although embryo to embryo variation in the size of these struts has been previously observed (Clouthier et al., 2003; Ruest and Clouthier, 2009). When only partially present, the strut was seen as a bony nodule lying in between the pterygoid and the middle ear (Clouthier et al., 2003; Ruest and Clouthier, 2009) (Fig. 6N).

**Fig. 7.**
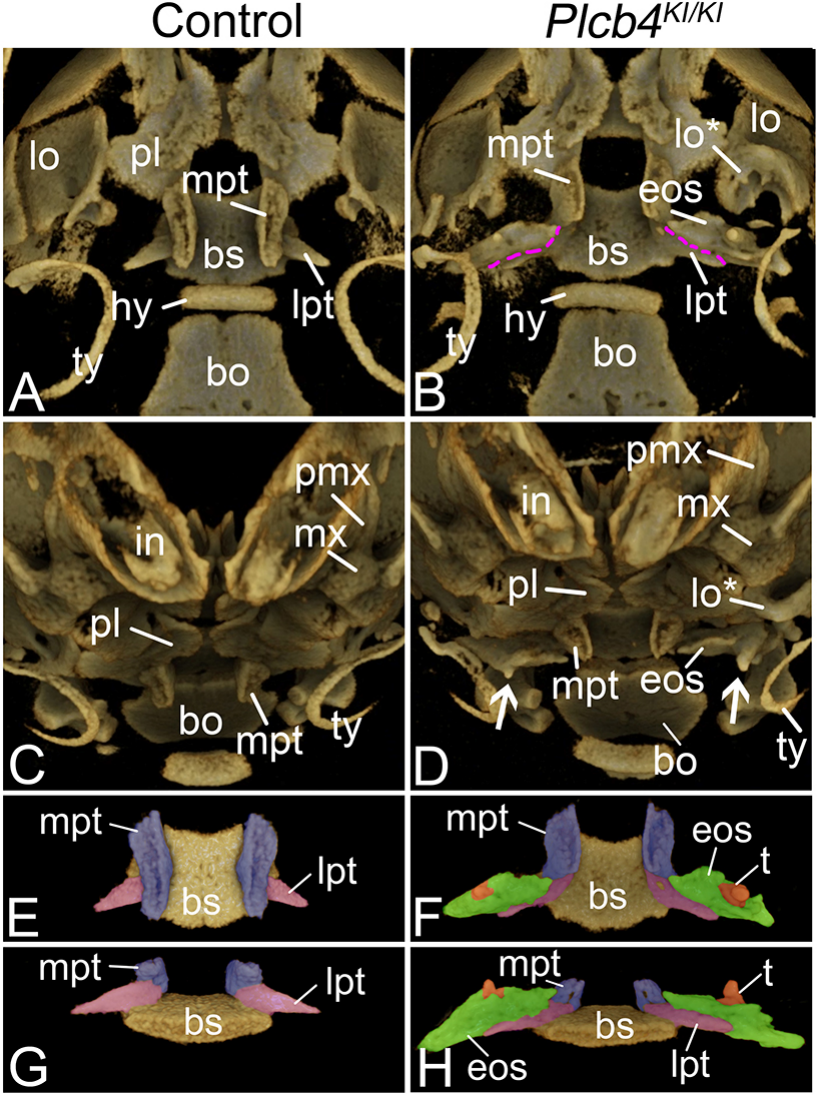
**Ectopic bone formation in the posterior palate of CRISPR embryos.** (A-H) µCT analysis of the skull in E18.5 control (A,C,E,G) and *Plcb4^KI/KI^* (B,D,F,H) embryos. (A-D) Ventral (A,B) and frontal (C,D) views of embryos. Purple dashed lines indicate the fusion point of the lateral pterygoid (lpt) and the ectopic osseous strut (eos). White arrows in D indicate tooth-like projections (t) on the ectopic osseous strut. (E-H) Isolated ventral (E,F) and frontal (G,H) views of the digitally isolated basisphenoid (bs)/pterygoid bone complex. The medial pterygoid (mpt) is pseudo-colored purple, the lateral pterygoid is pseudo-colored pink, the ectopic osseous strut is pseudo-colored green and the tooth-like structures are pseudo-colored orange. bo, basioccipital bone; hy, hyoid; in, incisor; lo, lamina obturans; lo*,duplicated lamina obturans; mx, maxilla; pl, palatine; pmx, premaxilla bone; ty, tympanic ring bone.

To better visualize changes in the posterior oral cavity, control and *Plcb4^KI/KI^* embryos were scanned at high resolution by µCT after bone staining and removal of the mandible. Compared with control embryos (Fig. 7A,C), the lamina obturans (the intramembranous bone region of the alisphenoid) in *Plcb4^KI/KI^* embryos was partially duplicated (Fig. 7B,D), a finding previously observed in *Ednra^−/−^* embryos (Ruest et al., 2004). More striking was that the ectopic osseous struts in *Plcb4^KI/KI^* embryos had a distinct shape with similar features on either side of the skull (Fig. 7B,D,F,H; Movies 1 and 2), resembling posterior or quadrate processes of the pterygoid generally observed in the skull of many non-mammalian amniotes (Evans, 2008; Romer, 1956). Both struts were fused to the ventral surface of the lateral pterygoid wings, which themselves were displaced proximally on the basisphenoid (Fig. 7B,F,H) compared with control embryos (Fig. 7A,E,G). The medial pterygoids were also dysplastic (Fig. 7B,F,H) compared with control embryos (Fig. 7A,E,G). The osseous struts were flattened on their ventral surface and were beveled on the medial end closest to the pterygoid bone (Fig. 7D,F; Movies 1 and 2). Furthermore, prominent rounded bony projections emanated from the bone and extended ventrally (Fig. 7D,H), resembling pterygoid teeth variably observed in non-mammalian amniotes (Evans, 2008; Mahler and Kearney, 2006; Romer, 1956). These observations suggest that the ectopic osseous struts do not simply represent random ossification of mesenchyme but rather an atavistic change in this region that results in the formation of structures that have been lost in the class Mammalia (Hall, 1984; Smith and Schneider, 1998).

### Novel phenotype in a child with ARCND2

**Fig. 8.**
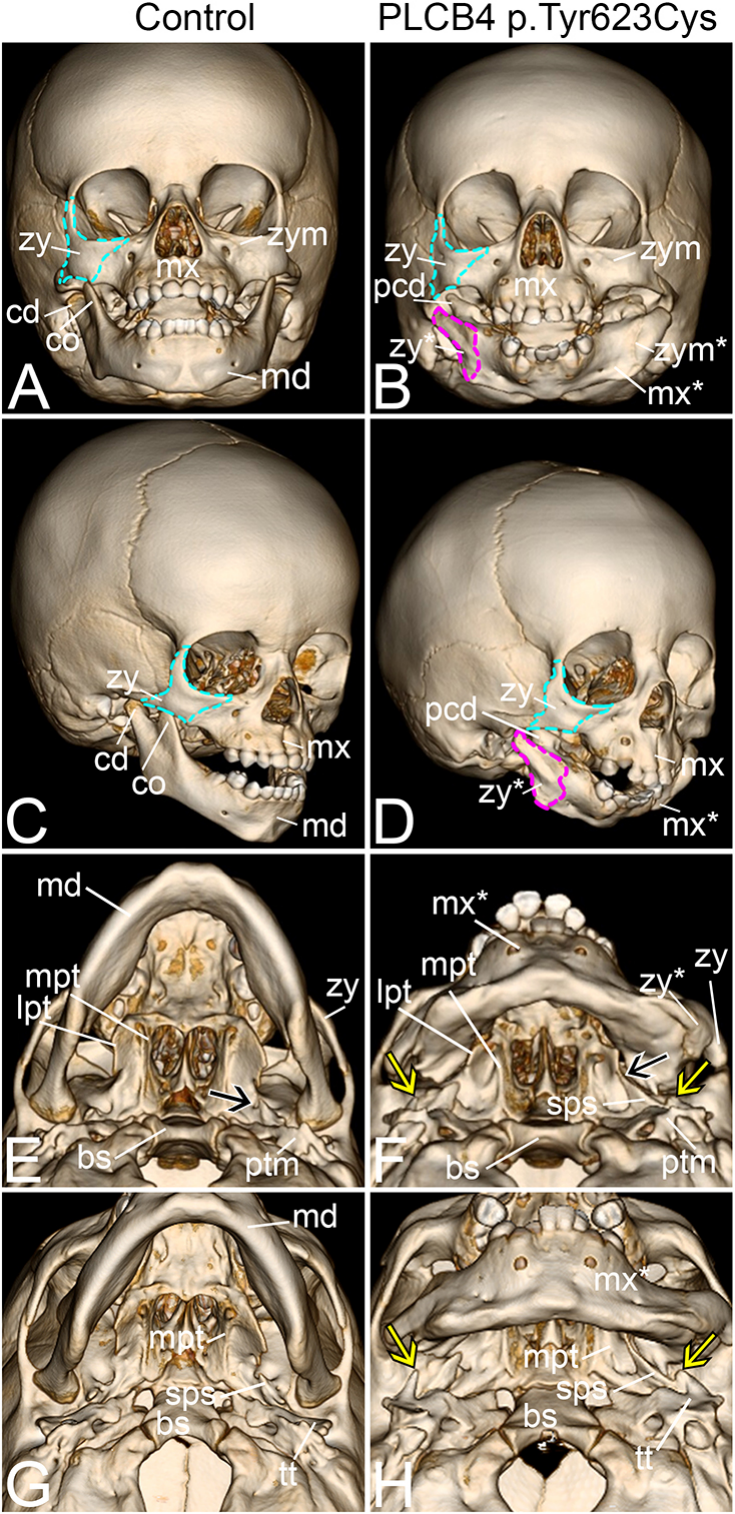
**Similar morphological changes observed in CRISPR embryos exist in the skull of a child with ARCND2.** (A-F) Three-dimensional renderings of CT scans from control (A,C,E,G) and a child with ARCND2 (B,D,F,H). (A-D) Frontal (A,B) and frontal oblique (C,D) views of the skull. The difference between A,B and C,D is 30° of head rotation. Cyan outlines in A-D indicate the zygoma (zy). Magenta outlines in B and D indicate the duplicated zygoma (zy*). (E-H) Inferior oblique (E,F) and inferior surface (G,H) views. The difference between E,F and G,H is 30° of head pitch. The black arrow in E indicates the foramen ovale. Yellow arrows in F and H indicate the dysplastic osseous projections that extend from the tympanic portion of the temporal bone (tt). The black arrow in F indicates a pterygospinous bar. bs, basisphenoid bone; cd, condylar process; co, coronoid process; lpt, lateral pterygoid; md, mandible; mpt, medial pterygoid; mx, maxilla; mx*, duplicated maxilla; pcd, pseudo-condylar process; ptm, petrous portion of temporal bone; sps, spinous process of the sphenoid bone; zp, zygomatic process of the temporal bone; zym, zygomaticomaxillary suture; zym*, duplicated zygomaticomaxillary suture.

The role for EDNRA signaling in jaw development is highly conserved in jawed vertebrates (Clouthier et al., 2013). To examine whether the skull changes observed in *Plcb4* CRISPR mouse embryos are also present in individuals with ARCND2, we analyzed 3D computed tomography (CT) scan data from a child with ARCND2 (age 11 months) carrying the PLCB4 p.Tyr623Cys variant (Rieder et al., 2012). Compared with a similarly aged unaffected child (Fig. 8A,C), the child with ARCND2 had a mandible that more closely resembled a mirror-image duplication of the maxilla, had no coronoid process and had a dysplastic pseudo-condylar process (Fig. 8B,D; Rieder et al., 2012), similar to duplications observed in mouse embryos that lack EDNRA signaling (Clouthier et al., 1998; Kurihara et al., 1994; Ozeki et al., 2004; Ruest et al., 2004; Yanagisawa et al., 1998). Furthermore, an anomalous laterally displaced bone is attached to the duplicated maxilla and extends proximally (outlined in magenta in Fig. 8B,D). This bone resembles the zygoma/jugal bone (outlined in aqua) in both control (Fig. 8A,C) and ARCND2 (Fig. 8B,D) individuals, suggesting that it represents a duplicated zygoma. This duplicated zygoma bone is fused to the duplicated maxilla through what we interpret as a duplication of the zygomaticomaxillary suture (Fig. 8B, similar to the fibrous suture observed in *Plcb4* CRISPR embryos; Fig. 6E,F).

We also carefully inspected the skull base in the child with ARCND2 at a location analogous to that where the ectopic osseous strut was observed in *Plcb4* CRISPR embryos (Fig. 6N,O, Fig. 7B,D,F,H). In a 3D CT scan from an unaffected similarly aged child, the lateral and medial pterygoid plates are well defined, with the foramen ovale clearly apparent (black arrow in Fig. 8E). In contrast, the pterygoid plates in the child with ARCND2 are mildly dysplastic (Fig. 8F) and a pterygospinous bar (Henry et al., 2020) bridges the foramen ovale (black arrow in Fig. 8F) before continuing towards and fusing with the spinous region of the sphenoid bone (Fig. 8F). When the child was older, this osseous bar enlarged and appeared distinct from the pterygospinous bar described as a human anatomical variant (not shown). In addition, an abnormal osseous process arises from the tympanic region of the temporal bone. This process contains abnormal bony projections pointed towards the dysplastic mandibular fossa bilaterally and towards the duplicated maxilla (yellow arrows in Fig. 8F,H). Although these findings do not completely phenocopy the ectopic osseous struts in hypomorphic *Ednra* mouse models or *Plcb4* CRISPR mouse embryos, it is reasonable to assume that this structure represents a phenotypic spectrum of an ectopic atavistic change that affects the region of the posterior roof of the oral cavity and/or skull base region that occurs when EDNRA signaling is reduced.

## DISCUSSION

ARCND2 is associated with variants in *PLCB4*, but neither causation nor underlying disease mechanism has been investigated. In this study, we have provided the first experimental evidence that ARCND2 variants encode PLCB4 mutants that exert dominant-negative interference on the EDNRA/Gq signaling pathway. Furthermore, we show that an ARCND2 disease allele is sufficient to cause jaw defects and changes in the posterior roof of the oral cavity in CRISPR mouse embryos. Analysis of skull changes in a child with ARCND2 found similar changes, illustrating that this overall approach can not only identify mechanisms behind human disease variants but may also help explain previously unreported or unrecognized clinical features.

### The dominant-negative mechanism of PLCB4 variants

ARCND2 PLCB4 variants affect amino acids that are essential for the PIP2 hydrolysis reaction mechanism (Ellis et al., 1998; Gresset et al., 2012), although why these mutations confer a dominant-negative effect rather than loss of function remains unclear. One explanation is that PLCB4 mutants trap PIP2 in a nonproductive enzyme-substrate complex. Given the low abundance of PIP2 in cells, accounting for only ∼1% of total membrane phospholipids (Czech, 2000; Harraz et al., 2020), PLCB4 mutants would sequester the available pool of PIP2 from wild-type PLCB isoforms, resulting in the diminished output of IP3 and DAG. This dominant-negative interference could be further exacerbated if the disease variants also impair the GTPase-activating protein (GAP) activity in addition to PIP2 hydrolysis activity of PLCB4. The GAP activity of PLCB accelerates the GTP hydrolysis rate of GTP-bound Gq/11, which facilitates rapid dissociation of Gq/11 from PLCB and subsequent reactivation of Gq/11 by a ligand-bound receptor. This on-off cycling mechanism, termed ‘kinetic scaffolding’ (Ross, 2011; Waldo et al., 2010), allows for rapid, repeated activation of PLCB to produce a temporally focused, high-amplitude signaling response. Thus, impaired GAP activity could potentially prolong the Gq/11-PLCB4 interaction and deplete the pool of Gq/11 that is available for both ligand-bound EDNRA and wild-type PLCB isoforms. Additional experiments are needed to determine whether these disease variants affect GAP activity and/or dissociation kinetics of the Gq/11-PLCB4 complex.

ARCND2 disease variants may also disrupt signaling dynamics controlled by preassembled GPCR-G protein-effector complexes. G proteins, including Gq, can exist as preassembled protein complexes with GPCRs, in which the receptor, G proteins and effectors exist in very close proximity to each other in the absence of ligand (Qin et al., 2011). This close proximity leads to the presence of ‘high-affinity’ receptors (Civciristov et al., 2018), in which the presence of a ligand leads to a rapid increase in signaling due to mechanisms that may include rebinding of ligand, increased ligand binding time and increased ligand sequestration/concentration (Dityatev and Schachner, 2006; Herbette et al., 1988; Hrabětová and Nicholson, 2004; Kane et al., 2008; Sargent et al., 1988; Sykes et al., 2014; Vargová and Syková, 2008; Vauquelin and Charlton, 2010). These high-affinity receptors can also respond to ultra-low ligand concentrations (Civciristov et al., 2018). The remaining receptors that are not part of a preassembled complex are considered low-affinity receptors and respond to high ligand concentrations (Civciristov et al., 2018). EDNRA has not been demonstrated to exist as part of a preassembled protein complex (Civciristov and Halls, 2019). However, if such preassembly occurred, it is possible that at least some EDNRAs could be stabilized in a preassembly complex containing dominant-negative PLCB4 (Staus et al., 2016). While these high-affinity receptors would not be able generate DAG, low-affinity receptors might still be able to generate DAG if the signaling complex used endogenous PLCB, potentially accounting for why we observe only partial dominant-negative signaling in our assays. In addition, signaling through these low-affinity receptors might explain why translocation of fluorescent DAG reporter to the membrane is so much slower in cells expressing PLCB4 variants (Fig. 2). Testing this possibility will require concentration-response and gene expression studies with ligand concentrations far below those used in this study.

The fact that DAG and IP3 could be generated in these models could explain why individuals with ARNCD develop defects in craniofacial elements but not in the cardiovascular outflow tract. Targeted deletion of *Ednra* in mice is neonatal lethal, with defects in both craniofacial and cardiovascular structures resulting from aberrant cranial and cardiac neural crest cell patterning, respectively. However, based on analysis of *Ednra^+/+^*↔*Ednra^−/−^* chimeric embryos (Clouthier et al., 2003), it appears that cardiovascular patterning requires only between 10 and 15% of normal EDNRA signaling, whereas craniofacial patterning requires 50% of normal signaling. Thus, although PLCB4 mutations reduce the overall activity and activation kinetics of other PLCB isoforms, the extent and rate of PIP2 conversion to DAG and IP3 is likely sufficient for normal cardiovascular development.

It is also possible that ARCND2 disease variants impact processes beyond craniofacial development, as PLCB4 can be activated by a multitude of GPCRs besides EDNRA through various mechanisms. *PLCB4* is predominantly expressed in the brain and retina, with lower expression in tissues and organs that also express Gq/11 (Uhlén et al., 2015; Wettschureck and Offermanns, 2005). *Plcb4^−/−^* mice have visual impairments and nerve defects (Han and Simon, 2011; Jiang et al., 1996), suggesting that PLCB4 dominant-negative variants have the potential to impact a wider range of processes than currently known. In addition, G15 and G16, which belong to the Gq/11 family, can couple promiscuously to a wide variety of GPCRs, and therefore can theoretically link Gs-, Gi/o- and G12/13-coupled receptors to PLCB activity (Offermanns and Simon, 1995). This could, in turn, expand functional consequences of ARCND2 variants to a group of cell types and tissues expressing G15 and G16 (Wilkie et al., 1991). Free Gβγ subunits from Gi/o-coupled receptors can also stimulate PLCB activity (Smrcka, 2008), although our assays did not test whether the ARCND2 variants could also interfere with Gβγ-mediated phospholipase activity. The functional consequence of ARCND2 variants may be negligible, however, because PLCB4 exhibits the lowest sensitivity to Gβγ relative to other PLCB isoforms (Lee et al., 1994). Although defects beyond craniofacial anomalies have not been reported in individuals with ARCND2, behavioral and phenotypic analysis of stable *Plcb4^R621H^* mice may reveal additional deficits caused by ARCND2 disease variants, allowing a better understanding of whether other receptor systems and signaling pathways are affected.

### Similarities of skull structures: a child with ARCND and F_0_ CRISPR mice

Although the human ARCND phenotype is highly variable (Gordon et al., 2013a,b; Nabil et al., 2020; Rieder et al., 2012), one stereotypical change is the apparent homeotic transformation of the mandible to maxilla-like structures (Clouthier et al., 2013; Gordon et al., 2014; Rieder et al., 2012). Analysis of our *Plcb4* CRISPR embryos has informed our investigation of CT scans from a child with ARCND2 to better define and understand the changes in facial structures. This has included identifying the abnormal lateral eminence on the pseudo-maxilla as a duplicated zygoma (jugal) bone, complete with a duplicated zygomaticomaxillary suture. This duplicated zygoma is the second homeotic transformation identified to date in individuals with ARCND2 and illustrates the highly conserved role of EDNRA signaling in establishing the identity of NCCs that give rise to lower jaw structures. Although the actual signaling disruptions leading to changes in proximal jaw development, including transformations, are not clear, it is interesting that proximal mandible defects in E18.5 *Erk2^fl/fl^;Wnt1-Cre* embryos (Parada et al., 2015) resemble those in E18.5 *Gnaq^fl/fl^;Gna11^−/−^;P0-Cre* embryos (Dettlaff-Swiercz et al., 2005). Examining the gene regulatory networks regulated by the Gq/ERK pathway in stable mouse lines carrying the PLCB4 p.Arg621His variant will be useful in dissecting these phenotypes further.

### EDNRA signaling and evolution of the posterior oral cavity

The ectopic osseous struts that extend from the pterygoid in our *Plcb4* CRISPR mouse embryos were also observed in *Ednra+/+*↔*Ednra^−/−^* chimeric mouse embryos and in *Ednra^fl/fl^;Wnt1-Cre* embryos, but not in *Ednra^−/−^* embryos (Clouthier et al., 1998, 2003; Ruest and Clouthier, 2009). This suggests that these structures form in animal models and in humans under conditions where EDNRA signaling is reduced but not absent. Our µCT scans have now shown that this strut has a highly organized structure that resembles the pterygoid (quadrate) processes of some extinct and extant amniotes, and also bears tooth-like structures. Generally, in non-mammalian amniotes, the pterygoid is characterized as a large triradiate structure that includes the posterior region of the palate and is one of the largest intramembranous bones of the skull. The posterolateral projections are the posterior or quadrate processes that typically contact (or nearly so) the median lamina of the quadrate (Romer, 1956; Versluys, 1936). In birds, the pterygoid is essentially composed of only these posterolateral projections (Marinelli, 1936). Similarly, the osseous projections observed in *Plcb4^KI/KI^* embryos also appear to articulate with the incus (Fig. 6O) (the mammalian equivalent of the quadrate). The posterior processes of the pterygoid are also present in synapsid lineages, including the precursors of mammals. Although they became gradually reduced among many cynodonts (Parrington and Westoll, 1940), they were retained in the Lower Jurassic mammaliaform *Morganucodon* (Kermack et al., 1981). Our results suggest that the developmental program for a more generalized non-mammalian palate remains ‘available’ at some level in mammals (Hall, 1984; Smith and Schneider, 1998) and it can be reactivated when EDNRA signaling drops below a certain threshold. Endothelin signaling long preceded the evolution of the jaw and was actually crucial for the evolution of the entire neural crest population (Square et al., 2020). Subsequent incorporation of new downstream targets of Ednra, like Hand genes, likely facilitated jaw evolution. Based on our results here, it is reasonable to suggest that the evolution of the Mammalia lower facial complex may have been mediated not only by the presence of EDNRA signaling in NCCs, but by increasing levels of EDNRA signaling in NCCs. This likely led to advantageous changes in lower jaw and middle ear structures (including the separation of the middle ear ossicles from the posterior palate/posterior oral cavity), with one consequence being repression of the posterior pterygoid processes.

Another puzzling question is the identity of the tooth-like projections on the posterior processes in *Plcb4* CRISPR mice and in a child with ARCND. Palatal dentition is common across a diverse array of non-mammalian amniotes (Romer, 1956). In squamate reptiles, these teeth are most often found on the pterygoid, though are variably present (Skawiński and Borczyk, 2017), largely on a lineage-specific basis (Mahler and Kearney, 2006). When present, they do not develop fully until after birth/hatching (Barahona and Barbadillo, 1997, 1998; Barahona et al., 1997), typically forming on the anterior and/or central regions of the pterygoid, although they can form as far posteriorly as the quadrate in some snakes. Their function is poorly understood, although in snakes with highly kinetic skulls, they participate in the specialized prey transport mechanism that facilitates the ingestion of large prey items (Cundall and Greene, 2000). In herbivorous iguanians, however, they have been ascribed roles in the processing of plant material (Montanucci, 1968). Similar to the posterior pterygoid processes, in *Plcb4* CRISPR embryos, these rounded projections likely represent a spectrum of palatal dentition that has become reactivated due to reduced EDNRA signaling. More-detailed analysis of these structures may help further elucidate their actual identity and developmental origin.

It is also interesting to consider how and when the struts and bony projections arise during NCC development. As described above, we believe that the struts and associated bony projections represent a developmental continuum that results from reduced EDNRA signaling within NCCs. From previous studies, we know that EDNRA signaling is required between E8.0 and E9.5 to pattern cranial NCCs in the mandibular region of arch one and more caudal arches (Ruest and Clouthier, 2009). Furthermore, only a reduction in EDNRA signaling during this time period results in strut formation (Ruest and Clouthier, 2009). This reduced signaling likely alters gene regulatory networks, permitting some NCCs to adopt an alternative developmental trajectory in the middle ear/posterior palate-oral cavity region and thus establishing a modified framework for NCC-derived membranous bone formation. Although the bones are ectopic (the strut and projections are not normally seen in mice), they are normally shaped (they resembles posterior pterygoid processes and pterygoid teeth). Subsequent membranous ossification of these processes and projections, like ossification of other facial structures in *Ednra^−/−^* embryos, does not require EDNRA signaling (Clouthier et al., 1998; Ruest et al., 2004). It will be interesting to compare NCC gene expression between wild-type and *Plcb4^R621H^* mouse embryos to see whether the basis of these atavistic changes can be uncovered. Taken together, these findings illustrate that while evolution of the craniofacial region is complex at both cellular and molecular levels, differences in bone structure between species may require only minor changes in common signaling networks, similar to how differences in BMP4 levels shape the beak of Darwin's finches (Abzhanov et al., 2004) or how a *ROR2* coding variant affects pigeon beak shape (Boer et al., 2021).

## MATERIALS AND METHODS

### Homology modeling

All computation-based modeling was performed using Biovia Discovery Studio 2020 (version 2.5, BIOVIA, Dassault Systèmes). Homology models were generated for human PLCB4 using NP_000924.3 as the target sequence for human PLCB4, and the structures for human PLCB2 (PDB: 2ZKM) (Hicks et al., 2008) or activated mouse Gq bound to human PLCB3 (PDB: 3OHM) (Waldo et al., 2010) as templates. Manual sequence alignment was performed, and the homology models were subsequently generated and further refined by energy minimization. The residues in the models were corrected for physiological pH, and loop refinement was performed. The most energy-favored model was retained for further consideration. The model was refined further using CHARMM (Brooks et al., 2009) and subjected to energy minimization (conjugate gradient, 1000 iterations) at a convergence of 0.001 kcal/mol using a Generalized Born implicit solvent model (Feig and Brooks, 2004). In the initial minimization, the protein backbone atoms were fixed, followed by a final minimization where all atoms were unfixed and restraints were removed.

### Sequence alignment

Amino acid sequence alignments were performed using Clustal Omega (Madeira et al., 2019) and the following sequences: human PLCB4 (NP_000924.3), human PLCB1 (NP_056007.1), human PLCB2 (NP_004564.2), human PLCB3 (NP_000923.1) and Zebrafish PLCB3 (ABM91767.1). Figures were then generated using ESPript 3.0 (Robert and Gouet, 2014).

### Plasmids and site-directed mutagenesis

All site-directed mutagenesis was performed using the QuickChange Lightning Site-Directed Mutagenesis Kit (Agilent Technologies). The human *PLCB4* (NM_182797) expression construct pCMV6-Myc-DDK-PLCB4 (Myc-FLAG-PLCB4) was purchased from Origene (RC217903). The disease variants were introduced into Myc-FLAG-PLCB4 by site-directed mutagenesis using the following primers for the indicated variant: p.Arg621His, 5′ CAAACGGCAAATGAGTCACATTTACCCCAAGGGAG 3′ and 5′ CTCCCTTGGGGTAAATGTGACTCATTTGCCGTTTG 3′; p.Tyr623Cys, 5′ CAAATGAGTCGCATTTGCCCCAAGGGAGGCCG 3′ and 5′ CGGCCTCCCTTGGGGCAAATGCGACTCATTTG 3′; p.Glu358Val, 5′ GTTGCAGATGTGTTGTACTTGACTGCTGGG 3′ and 5′ CCCAGCAGTCAAGTACAACACATCTGCAAC 3′; p.Asp360Val, 5′ CAGATGTGTTGAACTTGTCTGCTGGGATGGAAAAG 3′ and 5′ CTTTTCCATCCCAGCAGACAAGTTCAACACATCTG 3′; p. Pro838Ala, 5′ CGTGGATGCTTTATCAGATGCAAAGAAATTTCTCTC 3′ and 5′ GAGAGAAATTTCTTTGCATCTGATAAAGCATCCACG 3′.

The human *EDNRA* expression construct pCMV6-XL5-EDNRA was purchased from Origene (SC118901). The EDNRA p.Gln381Pro variant was previously generated (Pritchard et al., 2020). pEYFP-N1-Lyn_1-14_-Venus (hereafter referred to as Lyn-Venus) was a gift from Péter Várnai (Gulyás et al., 2017). pGL4.33 [*luc2P*/SRE/Hygro] (referred to as SRE:*luc2P*) was purchased from Promega (E1340). pcDNA3.1-HA-Gq was a gift from Nevin Lambert (Medical College of Georgia, University of Augusta). The pcDNA3.1-HA-Gq p.Glu209Leu mutant was generated by site-directed mutagenesis using the primers: 5′ GATGTAGGGGGCCTAAGGTCAGAGAG 3′ and 5′ CTCTCTGACCTTAGGCCCCCTACATC 3′.

pGFP-C1-PKCγ-C1A (referred to as GFP-C1A) was a gift from Tobias Meyer (Addgene 21205). To replace GFP with rLuc8, the GFP-C1-PKCγ-C1A fragment was first PCR amplified with ClaI and XbaI restriction sites on the 5′ and 3′ ends, respectively, using primers 5′ CCCATCGATATGGTGAGCAAGGGCGAGG 3′ and 5′ CTAGTCTAGATTACTTGTACAGCTCGTCCATGCCG 3′. The resulting amplicon was then cloned into a pCS2 vector linearized with ClaI and XbaI using T4 DNA Ligase (New England Biolabs). The resulting plasmid, pCS2-GFP-C1-PKCγ-C1A was then digested with ClaI and EcoRI to excise the GFP fragment, resulting in a linearized pCS2 acceptor plasmid containing PKCγ-C1A. Renilla luciferase 8 was PCR amplified from pEDNRA-rLuc8 (Pritchard et al., 2020) with primers 5′ GCAGGATCCCATCGATATGGCTTCCAAGGTGTACGACC 3′ and 5′ GTCGACTGCAGAATTCCTGCTCGTTCTTCAGCACGC 3′, and then cloned in-frame on the 5′ end of PKCγ-C1A using the In-Fusion Cloning Kit (Takara Bio), resulting in the plasmid pCS2-rLuc8-PKCγ-C1A (hereafter referred to as rLuc8-C1A).

### Cell culture and transfection

HEK293T (CRL-11268) and HEK293 (CRL-1573) cells were purchased from American Type Culture Collection (ATCC) and were thus not authenticated. Cells were tested and confirmed to be mycoplasma free. Cells were maintained in Dulbecco's Modified Eagle Medium (DMEM) (Corning) supplemented with 10% fetal bovine serum in a tissue culture incubator at 37°C and 5% CO_2_ in the absence of antibiotics. Transfections were performed with X-tremeGENE 9 (Roche), using a 3:1 ratio of X-tremeGENE 9 to plasmid DNA.

### Time-lapse imaging assay

HEK293T cells were seeded at 6×10^5^ cells per dish in glass bottom dishes (MatTek) coated with poly-D lysine (100 μg/ml, Millipore Sigma) and co-transfected with EDNRA, GFP-C1A and wild-type PLCB4 or PLCB4 p.Arg621His at a 2:1:1 ratio (500:250:250 ng). After 6 h, growth media was replaced with serum-free DMEM and incubated overnight. 24-36 h after transfection, time-lapse imaging was performed using a Leica TCS SP5 confocal microscope and 63× oil objective. Images were taken in 30 s intervals for a total of 12 min. First, basal reporter activity was imaged for 2 min, then stimulated with EDN1 and imaged for 10 min. To prevent subjective bias, the experimental conditions for the acquired images were masked for subsequent analysis. Individual cells were chosen for analysis only if the fluorescence intensity of the reporter was below saturation and the cell boundaries were clearly defined. To quantify the change in membrane fluorescence in individual cells, we identified the first time-lapse frame in which translocation was observed. The plot profile tool in Image J was then used to draw a line across the cell to obtain the average fluorescence intensity in the cytoplasm (F_cyto_) and plasma membrane (F_pm_). The following equation was then used to determine the relative change in membrane fluorescence: ΔF_pm_=(F_pm_−F_cyto_)/F_cyto_ (Oancea et al., 1998). Gaussian fitting of the histogram was performed using Prism (GraphPad), with statistical significance determined by chi-square test for trends in Prism.

### Bystander bioluminescence resonance energy transfer (BRET) assay

HEK293T cells were seeded at 1.2×10^6^ cells per well in a six-well tissue culture dish and co-transfected with the vectors expressing EDNRA, rLuc8-C1A, Lyn-Venus and wild-type PLCB4 or the indicated PLCB4 mutants at a 4:1:1:2 ratio (1000:250:250:500 ng). After 24 h, growth medium was replaced with serum-free DMEM and, 12 h later, cells were dissociated in EDTA-free PBS (Thermo Fisher Scientific), pelleted by centrifugation (500 ***g***) and resuspended in reaction buffer (Tyrode's Salts with 0.1% glucose, Millipore Sigma). Treatment with the Gq/11 inhibitor YM-254890 (Cayman Chemical) was performed by resuspending cells in reaction buffer containing the indicated concentration of YM-254890 and incubating for 5 min before proceeding to the next step. 1.2×10^5^ cells were dispensed to individual wells of an opaque 96-well plate (Perkin Elmer, 6005299) and 10 µM coelenterazine *h* (Nanolight Technology) was added to each well. After a 3 min incubation, assays were performed using a Synergy 2 microplate reader (Biotek) equipped with emission filters for rLuc8 (485/20 nm) and Venus (528/20 nm). Basal BRET was measured for 1 min, then EDN1 (1 μM final concentration) was added to wells and the response was measured for 5 min. BRET was monitored by detecting 485/20 nm and 528/20 nm emissions every 2 s using automatic filter switching. Normalized BRET values were calculated by dividing the acceptor emission (Venus; 528/20 nm) by the donor emission (rLuc8; 485/20 nm). Normalized BRET values were then converted to ΔBRET values using the equation: ΔBRET=BRET_t_−BRET_basal_/BRET_basal_, where BRET_t_ is the BRET value at any given time point, and BRET_basal_ is the average BRET value of the 1-min basal recording prior to EDN1 addition. ΔBRET traces were fit to a single-phase exponential curve to quantify the maximum response values. Each experimental condition was performed at least three times in triplicate wells. Curve fitting and statistical analysis were conducted using Prism (GraphPad).

### Transcriptional reporter assay

HEK293 cells were seeded at 5×10^5^ cells per well in a 12-well tissue culture dish and co-transfected with SRE:*luc2P*, Gq p.Gln209Leu and wild-type PLCB4 or the indicated PLCB4 mutant at a 2:1:1 ratio (500:250:250 ng). After 6 h, growth media was replaced with serum-free DMEM. 12 h later, cells were dissociated with EDTA-free PBS, pelleted by centrifugation (500 ***g***), and resuspended in serum-free DMEM. 60,000 cells were dispensed to individual wells in an opaque 96-well plate, and an equivalent volume of ONE-Glo Luciferase substrate (Promega) was added to each well. After a 3 min incubation, luminescence was measured using a Synergy 2 microplate reader. Each experimental condition was performed at least three times in triplicate wells. Statistical analysis was conducted using Prism.

### Immunofluorescence and image acquisition

HEK293T cells were seeded at 5×10^5^ cells per well on poly-D lysine-coated (100 μg/ml) glass coverslips in a six-well tissue culture dish and transfected with wild-type PLCB4 or the indicated PLCB4 mutant. After 36 h, cells were fixed in 4% paraformaldehyde in PBS for 15 min at room temperature, permeabilized with 0.1% Triton X-100 in PBS for 10 min at room temperature, then incubated for 1 h at room temperature in blocking buffer (5% goat serum and 0.1% Tween 20 in PBS). Cells were then incubated with a mouse anti-Myc (9e10) primary antibody (Thermo Fisher Scientific, MA1-980) in blocking buffer overnight at 4°C. Cells were washed three times for 1 h each with 0.1% Tween 20 in PBS and then incubated with Alexa Fluor 488 goat anti-mouse IgG1(γ1) secondary antibody (Thermo Fisher Scientific, A21121) for 1 h at room temperature. Cells were then washed and mounted in ProLong Gold antifade reagent (Thermo Fisher Scientific). Cells were imaged using a Leica TCS SP8 confocal microscope and 63× oil objective. Images were taken as *z*-stacks and are presented as maximum projection images.

### Western blot analysis

Cells were lysed in radioimmunoprecipitation assay (RIPA) buffer containing 1× Complete Protease Inhibitor (Roche) and cleared by centrifugation. Cleared lysates were mixed with Laemmli buffer [25% glycerol, 5% β-mercaptoethanol, 2% sodium dodecyl sulfate, 0.01% Bromophenol Blue, 62.5 mM Tris (pH 6.8)] and boiled for 10 min. Lysates were then resolved with 10% SDS-PAGE and transferred to Immobilon PVDF membrane (Millipore Sigma). Membranes were incubated in blocking buffer [5% milk in TBST: 25 mM Tris (pH 7.2), 150 mM NaCl, 2.7 mM KCl, 0.1% Tween 20] for 1 h at room temperature, and then with mouse anti-FLAG (M2) (Millipore Sigma, cat. F1804) or mouse anti-alpha tubulin (12G10) (Developmental Studies Hybridoma Bank) primary antibody in blocking buffer at 4°C overnight. Membranes were washed with TBST and incubated with HRP-conjugated anti-mouse (Cell Signaling) secondary antibody for 1 h at room temperature. Membranes were then washed with TBST, incubated with SuperSignal West Pico Chemiluminescent Substrate (Thermo Fisher Scientific) and imaged with a ChemiDoc Imaging System (Bio-Rad Laboratories). Band intensity was quantified using the Image Lab software (Bio-Rad Laboratories). Quantified band intensities for FLAG were normalized to α-tubulin (loading control), and normalized values were then expressed as a percentage of wild-type PLCB4. Statistical analysis was conducted using Prism.

### Mouse *Plcb4* CRISPR/Cas9 genome editing

All experiments involving mice were approved by the Institutional Advisory and Use Committee at The Jackson Laboratory (Protocol 20028).

#### Design and sourcing of guide RNA and donor oligos

Candidate guide RNA (gRNA) sequences for CRISPR/Cas9 editing were selected on the basis of off-target and efficiency scores, as well as proximity to the orthologous human variant site. Based on these criteria, the guide Plcb4-R621H-Rev 5′ GGCCTCCCTTGGGGTAAATGCGG 3′ (reverse strand) was selected as the gRNA sequence for *Plcb4*. This sequence was used to create the unique CRISPR RNA (crRNA), which also contains a 16-nucleotide complementary sequence to the common tracrRNA (trRNA). A 120 bp donor oligonucleotide [single-stranded DNA (ssDNA)] harboring the *Plcb4* mutation c.G1862A (second bolded underlined nucleotide) was designed to create the p.Arg621His variant GTTCTCTGTTGTGCGTTCGCCTTGGCTGCTCTTGGATTTCCTTAATCAGTTACCCAGTTACAATAAGCGACAAATGAG**T**C**A**CATTTACCCCAAGGGAGGCCGAGTTGATTCCAGTAATT (forward strand). A silent mutation in the PAM sequence (first bolded underlined nucleotide) was included to reduce potential recutting. The crRNA, trRNA and ssDNA were sourced from Integrated DNA Technologies (IDT).

#### Guide RNA annealing and ribonucleoprotein complex formation

Plcb4-R621H crRNA was annealed with trRNA following IDT Alt-R System protocols. Briefly, both components were resuspended at 100 μM in IDT Duplex Buffer, combined in equal amounts, heated to 95°C for 5 min and allowed to cool passively to room temperature. Following this annealing step, the concentration of annealed guide RNA was assayed by NanoDrop. The CRISPR:tracr guide RNA hybrid was complexed with Alt-R Cas9 at 37°C for 15 min in a thermocycler.

#### Guide RNA testing in blastocyst culture

Prior to Plcb4-R621H F_0_ experiments, the editing efficiency of the gRNA and ssDNA donor were first tested *ex vivo*. Zygotes were harvested from naturally mated super-ovulated C57BL/6NJ females and electroporated with the ssDNA donor (1000 ng/μl) and the ribonucleoprotein complex containing Alt-R Cas9 nuclease (250 ng/μl) and gRNA (300 ng/μl). Following electroporation, zygotes were cultured in Sydney Cleavage Medium (COOK Medical) at 37°C in 5% CO_2_ in a benchtop incubator (COOK Medical). After 96 h, individual blastocysts were collected and lysed in DNA extraction buffer (25 mM NaOH and 0.2 mM EDTA) at 95°C for 15 min, and then neutralized with an equal volume of 40 mM Tris HCl. The extracted DNA was used to PCR amplify the editing site of the *Plcb4* genomic locus using the primers 5′ CAGACGTACATGCGTTGTTTCC 3′ and 5′ TTTCACATGGCAGCTTCCTTTA 3′, generating a product size of 423 bp. The amplification product was assessed for editing efficiency by Sanger sequencing. The selected gRNA sequence and ssDNA donor yielded a sufficient editing rate (Fig. S2) and were subsequently used to generate F_0_ mice.

#### Zygote electroporation and F_0_ embryo harvest

All mouse procedures were conducted according to national and international guidelines (AALAC and IACUC) and have been approved by The Jackson Laboratory Animal Care and Use Committee. For electroporation, zygotes from C57BL/6NJ mice were harvested and placed in a 20 µl droplet comprised of 10 μl TE buffer with ssDNA donor (2000 ng/μl), Alt-R Cas9 nuclease (500 ng/μl), gRNA (600 ng/μl) and 10 μl Opti-MEM reduced serum media (Millipore Sigma). This mixture was transferred to an electroporation cuvette (Harvard Apparatus) with a 1 mm gap electrode. Using a BTX ECM830 Square-pulse Electroporator (Harvard Apparatus), embryos were electroporated with six 3 ms pulses of 30 V at 100 ms intervals. Electroporated zygotes were immediately implanted in pseudopregnant dams via oviduct transfer, designating the transfer/implantation date as embryonic day 0.5 (E0.5). Embryos were then collected at E18.5 and fixed in 4% paraformaldehyde.

#### Genotyping of CRISPR/Cas9 F_0_ embryos

Genomic DNA was extracted from embryonic tail tips and the editing site of the *Plcb4* locus was PCR amplified using the primers described above that were used to test editing efficiency. PCR products were then analyzed by Sanger sequencing. Sequence traces were first manually curated for preliminary scoring of homology-directed repair (HDR) and non-homologous end joining (NHEJ) events. Sequences were also analyzed using the Inference of CRISPR Editing (ICE) deconvolution tool from Synthego (https://ice.synthego.com; Synthego Performance Analysis, ICE Analysis. 2019. v2.0.).

### Skeletal analysis

E18.5 embryos were collected, fixed and stained with Alizarin Red and Alcian Blue to visualize bone and cartilage, respectively, as previously described (Ruest et al., 2004).

### µCT analysis of CRISPR/Cas9 F_0_ embryos

Fixed F_0_ embryos were incubated in stabilization buffer (0.1% bis-acrylamide, 4.5% acrylamide, 4% paraformaldehyde, 0.3% VA044 and 0.05% saponin) for 72 h at 4°C. Samples were then flushed with nitrogen gas and polymerized at 37°C for 3 h. After removal from cured hydrogel, embryos were embedded in 1% agarose in 5 ml polypropylene transfer tubes (Fisher Scientific) and scanned with a Skyscan 1172 microCT with a 0.5 μm aluminum filter (Bruker BioSpin) using the following parameters: 13.49 µm resolution, 100 kV, 100 µA, 4400 ms exposure, 0.3° step size, 360° rotation and nine-frame averaging.

Additional embryos that had been used for skeletal staining (above) were also imaged using µCT. These embryos were placed individually in polypropylene cryovial tubes filled with PBS and scanned with a Skyscan 1275 microCT (Bruker BioSpin) using the following parameters: 8.5 µm resolution, 40 kV, 200 µA, 45 ms exposure, 0.3° rotation step, 180° imaging and four-frame averaging. Raw images from all scans were reconstructed using NRecon software (Bruker BioSpin).

Reconstructed scan data were imported into Drishti volume exploration software (version 2.63) (Limaye, 2012) for 3D rendering. Rendering settings were optimized for visualization and phenotypic assessment of mineralized tissues. To visualize the pterygoid/basisphenoid complex in isolation, two operations were performed on the Drishti-rendered volumes. Initially clip planes were used to exclude the bulk of the cranial bone around the complex. The MOP-carve function was then used to remove remaining bone from around the complex. To make rotational movies of the complexes to aid inspection, the Keyframe Editor function of Drishti was employed. For this, a new rotational axis was assigned for each volume and the initial keyframe set to mark the starting view of the rotation. The desired end of the rotation was set using the Bricks Editor function and a second keyframe set. All interpolated keyframes between the starting and ending keyframe were then saved as an image sequence in png format. Image sequences were then opened in Adobe Photoshop 2020 and rendered in mp4 format. Selected images from the renderings were saved and optimized for contrast, color and background using Adobe Photoshop.

### Human CT scanning

All studies were approved by the University of Washington Institutional Review Board (10926). Maxillofacial computed tomography scans were obtained for clinical purposes from a child with ARCND2 and an age-matched child with normal skull base anatomy scanned for an unrelated clinical indication. Scans were performed using a Toshiba Aquilion 16, GE Lightspeed VCT or Siemens Definition CT scanner. All scanners are helical, multi-slice CT units. Scans were performed with 0.5 s rotation time, no gantry tilt, pitch varying from 0.55 to 1 and tube voltage between 100 and 120 kVp. The Siemens scanner used iterative reconstruction, tube current modulation and automatic kVp selection. CT images were deidentified and volume-rendered 3D reconstruction performed using a Siemens *syngo*.via workstation.

## Supplementary Material

Supplementary information
