# Supplementary material for: Auriculocondylar syndrome 2 results from the dominant-negative action of PLCB4 variants
Source: Dis Model Mech. 2022 Apr 29;15(4):dmm049320. doi: 10.1242/dmm.049320 (PMC9066496; doi:10.1242/dmm.049320)
Supplement: Supplementary information [file dmm-15-049320-s1.pdf]

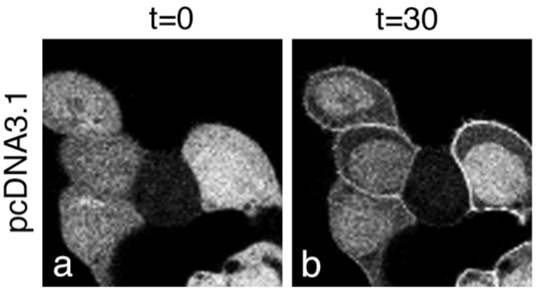

Fig. S1. Transfection of exogenous PLCB4 is not necessary for EDN1 to stimulate a DAG reporter response. Cells were transfected with GFP-C1, EDNRA, and empty vector (pcDNA3.1) and imaged before (0 seconds; t=0) and after addition of EDN1. Cytoplasm to membrane translocation of GFP-C1 was observed within the first imaging frame following EDN1 addition (30 seconds; t=30).

Disease Models & Mechanisms • Supplementary information

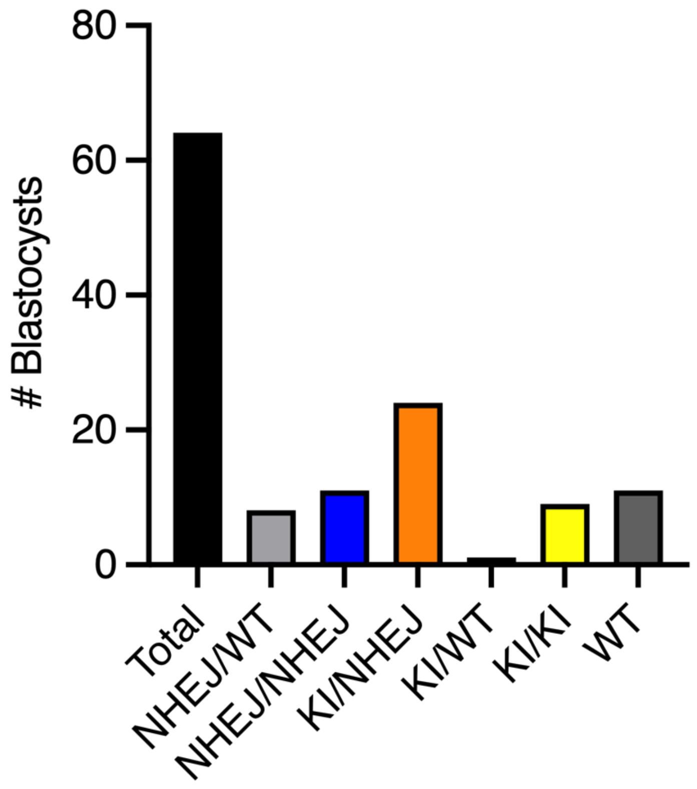

Fig. S2. Types of targeting observed in *Plcb4* CRISPR blastocysts. After electroporation, 64 blastocysts were collected and genomic DNA submitted for Sanger sequencing. The bars represent the type of editing that was detected.

Disease Models & Mechanisms • Supplementary information

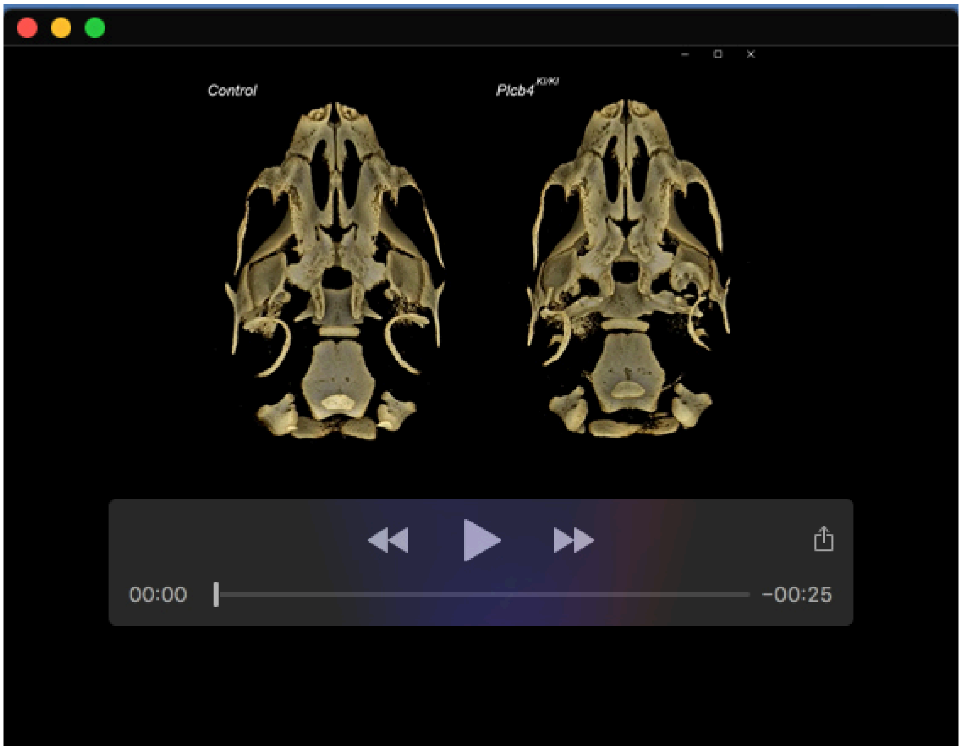

Movie 1. Rotating μCT images of E18.5 control (left) and *Plcb4*$^{KI/KI}$ (right) embryos.

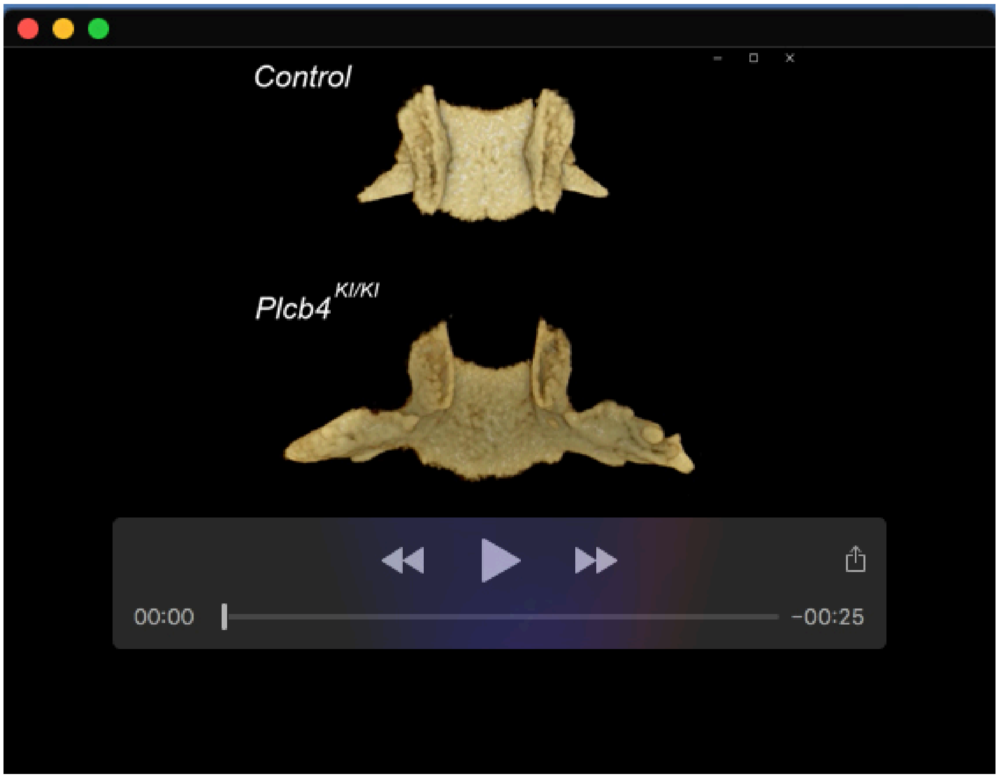

Movie 2. Rotating μCT images of the digitally-dissected basisphenoid/pterygoid complex from E18.5 control (top) and *Plcb4*$^{KI/KI}$ (bottom) embryos.

Disease Models & Mechanisms • Supplementary information
